# Vertebrate lonesome kinase modulates the hepatocyte secretome to prevent perivascular liver fibrosis and inflammation

**DOI:** 10.1242/jcs.259243

**Published:** 2022-04-12

**Authors:** Sophia Pantasis, Juliane Friemel, Salome Mirjam Brütsch, Zehan Hu, Sabrina Krautbauer, Gerhard Liebisch, Joern Dengjel, Achim Weber, Sabine Werner, Mattia Renato Bordoli

**Affiliations:** 1 Department of Biology, Institute of Molecular Health Sciences, Swiss Federal Institute of Technology ETH, Otto-Stern Weg 7, CH-8093 Zurich, Switzerland; 2 Department of Pathology and Molecular Pathology, University of Zurich and University Hospital Zurich, Rämistrasse 100, CH-8091 Zurich, Switzerland; 3 Department of Biology, Université de Fribourg, Chemin du Musée 10, CH-1700 Fribourg, Switzerland; 4 Department of Clinical Chemistry and Laboratory Medicine, University Hospital Regensburg, Franz-Josef-Strauss-Allee 11, DE-93053 Regensburg, Germany

**Keywords:** Collagen, Fibrosis, Liver, Secreted kinase, VLK, *Pkdcc*

## Abstract

Vertebrate lonesome kinase (VLK) is the only known extracellular tyrosine kinase, but its physiological functions are largely unknown. We show that VLK is highly expressed in hepatocytes of neonatal mice, but downregulated during adulthood. To determine the role of VLK in liver homeostasis and regeneration, we generated mice with a hepatocyte-specific knockout of the VLK gene (*Pkdcc*). Cultured progenitor cells established from primary hepatocytes of *Pkdcc* knockout mice produced a secretome, which promoted their own proliferation in 3D spheroids and proliferation of cultured fibroblasts. *In vivo*, *Pkdcc* knockout mice developed liver steatosis with signs of inflammation and perivascular fibrosis upon aging, combined with expansion of liver progenitor cells. In response to chronic CCl_4_-induced liver injury, the pattern of deposited collagen was significantly altered in these mice. The liver injury marker alpha-fetoprotein (AFP) was increased in the secretome of VLK-deficient cultured progenitor cells and in liver tissues of aged or CCl_4_-treated knockout mice. These results support a key role for VLK and extracellular protein phosphorylation in liver homeostasis and repair through paracrine control of liver cell function and regulation of appropriate collagen deposition.

This article has an associated First Person interview with the first author of the paper.

## Abstract (precis)

**Summary:** The secreted protein kinase VLK is released from hepatocytes and protects the liver from perivascular fibrosis and inflammation.

## INTRODUCTION

Vertebrate lonesome kinase [VLK, also known as protein kinase domain containing, cytoplasmic (PKDCC)] is the only known tyrosine kinase that is secreted and acts in the extracellular environment (Bordoli et al., 2014). It phosphorylates a wide range of proteins in the secretory pathway and extracellular space on tyrosine residues located within specific functional domains (Bordoli et al., 2014). VLK is highly abundant in α-granules of platelets, from which it is released upon degranulation, but it is also expressed in many other cell types (Bordoli et al., 2014). It plays a crucial role in normal platelet function and modulates thrombus formation and stimulus-dependent platelet activation (Revollo et al., 2021). The enzyme is able to phosphorylate co-released substrates in a cell-free environment using endogenous ATP (Bordoli et al., 2014). Global knockout of the gene encoding VLK (*Pkdcc*) in mice caused early postnatal death and revealed an involvement of the protein in skeletal, craniofacial and pulmonary development (Imuta et al., 2009; Kinoshita et al., 2009). *Pkdcc* expression increased in the absence of hedgehog signaling, and VLK was reported to synergize with the hedgehog effector glioma-associated oncogene 3 (Gli3) to coordinate the kinetics of chondrocyte differentiation (Probst et al., 2013). Conversely, VLK negatively regulated hedgehog signaling by interacting with the extracellular domain of Smoothened, thereby leading to its lysosomal degradation (Kim et al., 2020). Furthermore, VLK-dependent phosphorylation of repulsive guidance molecule B was shown to modulate Wnt3a activity, which is crucial for the formation of neuronal circuitries (Harada et al., 2019). Despite its critical role in development and the identification of a broad range of extracellular substrates *in vitro*, its expression and function in most organs has not been described. In addition, there is no documented role of VLK in tissue repair or fibrosis, which are characterized by major changes in extracellular matrix (ECM) proteins, some of which are targets of VLK (Bordoli et al., 2014).

Here, we analyzed VLK function in the liver, which has a remarkable regenerative capability that is unique amongst mammalian organs (Michalopoulos, 2007). This feature is vital due to the liver's numerous metabolic and detoxifying functions. In response to liver resection, exposure to various toxins or viruses or following autoimmune reactions, the liver has different ways to repair the damaged tissue. Hepatocytes and cholangiocytes are highly efficient in replicating and rebuilding the liver after a variety of injuries (Malato et al., 2011). However, severe acute or chronic injuries require the activation of alternative mechanisms, including transdifferentiation of hepatocytes into biliary-like cells (Michalopoulos and DeFrances, 2005; Gadd et al., 2020) or differentiation of stem cells from the biliary epithelium into hepatocytes (Español-Suñer et al., 2012; Raven et al., 2017; Deng et al., 2018; Trivedi et al., 2021). In these situations, the regenerative capacity of the liver is frequently insufficient, resulting in acute liver failure or development of liver fibrosis and cirrhosis (Biagini and Ballardini, 1989; Lee et al., 2015). Liver regeneration requires coordinated dynamic ECM remodeling. Aberrant ECM deposition (especially of collagens) and uncontrolled ECM rearrangement are key features of liver fibrosis and cirrhosis (Biagini and Ballardini, 1989; Lee et al., 2015). Therefore, identification of the genes and proteins, which control matrix remodeling under physiological and pathological conditions, is of utmost importance. Here we show that hepatocyte-derived VLK protects the liver from abnormal collagen deposition during aging and toxin-induced fibrosis, most likely through modulation of the hepatocyte secretome.

## RESULTS

### VLK is predominantly expressed in hepatocytes of neonatal mice and in cholangiocytes of adult mice

To determine a potential role of VLK in the liver, we first analyzed published RNA sequencing data (Ding et al., 2016), which revealed that *Pkdcc* is expressed in hepatocytes and in non-parenchymal liver cells of adult mice.

**Fig. 1.**
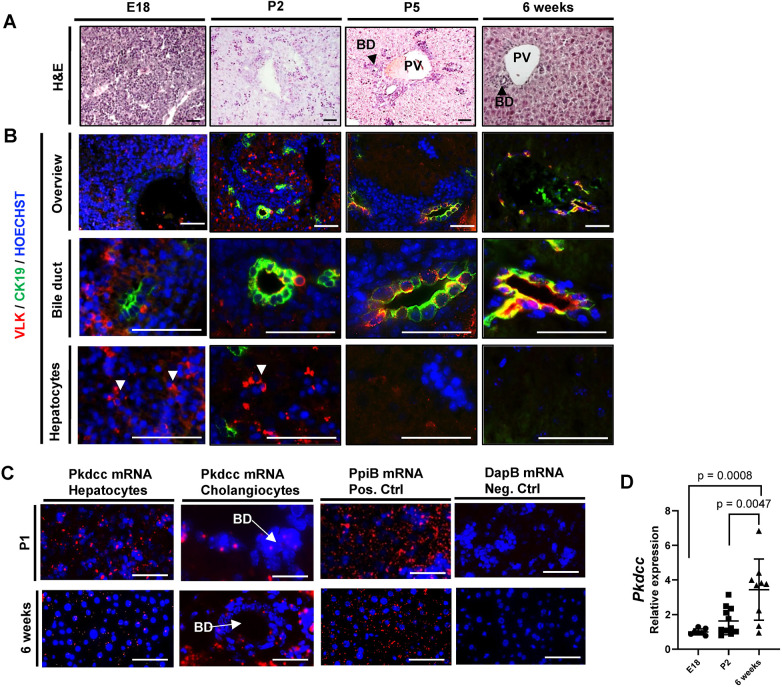
**VLK is expressed in a stage-specific manner in hepatocytes and cholangiocytes of mouse liver.** (A,B) Representative photomicrographs of mouse liver sections at the indicated developmental stages stained with Hematoxylin and Eosin (H&E) (A) or analyzed by immunofluorescence for VLK (red) and CK19 (green) (B). Arrowheads in B indicate cell clusters of VLK-positive cells. Nuclei were counterstained with Hoechst 33342 (blue). PV, portal vein; BD, bile duct. *n*=6. Scale bars: 100 μm. (C) *Pkdcc* mRNA in mouse liver at the indicated ages, visualized by RNA-Scope *in situ* hybridization (red). *PpiB* (red) and *DapB* mRNAs were used as positive and negative controls, respectively. Nuclei were counterstained with Hoechst. *n*=5. Scale bars: 100 μm. (D) RNA from whole liver of wild-type mice at the indicated ages was analyzed for *Pkdcc* expression relative to *Rps18* expression by qPCR. *n*=9 mice per time point. Graph shows mean±s.d. Mean expression at E18 was set to 1. Statistical significance was assessed by ordinary one-way ANOVA (Tukey's multiple comparisons test).

Histological analyses and immunofluorescence staining of liver tissue from mice at embryonic day 18 (E18), postnatal day 2 (P2), postnatal day 5 (P5) and at 6 weeks of age revealed predominant VLK expression in a subset of clustered cells at E18 and P2. Their distribution and nuclear morphology, as well as staining of serial sections with antibodies against VLK and the hepatocyte marker albumin (ALB), suggest that most of the VLK-positive cells are hepatocytes (Fig. 1A,B; Fig. S1A). In adult mice, VLK staining was most pronounced in cholangiocytes, the epithelial cells lining the bile ducts, as confirmed by co-localization with the cholangiocyte markers cytokeratin 19 (CK19, also known as KRT19) (Fig. 1B) and osteopontin (OPN, also known as SPP1) (Fig. S1B). The predominant expression of VLK in cholangiocytes of adult liver was confirmed with two additional VLK peptide antibodies (i.e. VLK 289 and VLK 404) (Fig. S1C). Their specificity was demonstrated by immunofluorescence staining of human embryonic kidney 293 cells expressing simian vacuolating virus 40 (SV40) large T antigen (HEK 293T cells) transfected with a VLK expression plasmid (Fig. S1D).

RNA-Scope *in situ* hybridization confirmed the increase in *Pkdcc* expression in cholangiocytes of adult mice compared with P1 mice, and the concomitant downregulation in hepatocytes during postnatal liver development, although *Pkdcc* mRNA was still detectable in this cell type in adult mice (Fig. 1C). The specificity of the probe was validated in HEK 293T cells overexpressing recombinant VLK (Fig. S1E). *Pkdcc* expression levels in whole liver increased in 6-week-old mice compared with postnatal mice as determined by quantitative real-time PCR (qPCR) (Fig. 1D).

### VLK modifies the mitogenic properties of the hepatocyte and cholangiocyte secretome

**Fig. 2.**
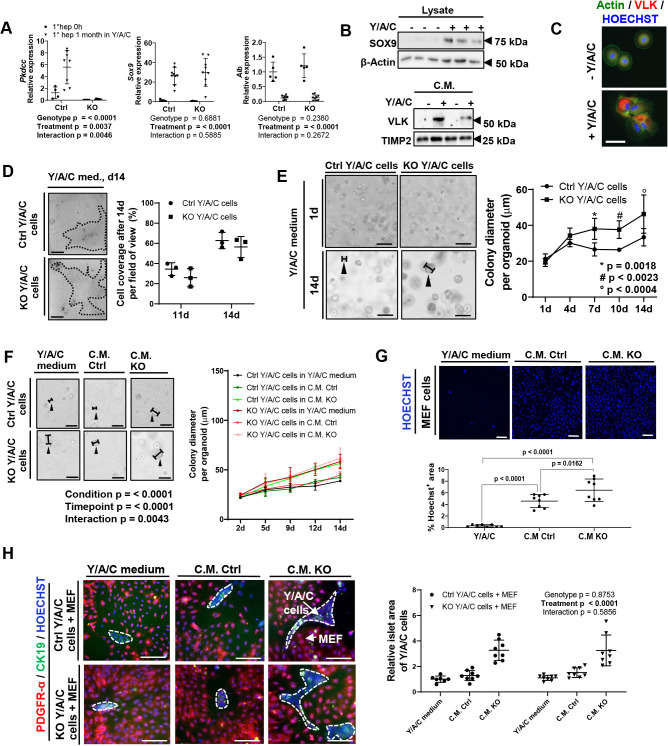
**Cultured hepatocyte progenitor cells secrete VLK, which affects their proliferation.** (A) Primary hepatocytes (1°hep) from Ctrl or KO mice were cultured in Y/A/C-supplemented medium for one month. Prior to (0 h) and following this culture period, RNA was analyzed by qPCR for *Pkdcc*, *Sox9* and *Alb* expression relative to *Rps18* expression. Combined results from two independent experiments are shown, each dot represents cells from one mouse. Ctrl 0 h: *n=*4; Ctrl one month: *n=*8; KO 0 h: *n=*5; KO one month: *n=*9. Mean expression levels in Ctrl mice were set to 1. *P*-values are given for the following comparisons: Genotype, control and KO; Treatment, primary hepatocytes directly after isolation and primary hepatocytes cultivated for one month in Y/A/C medium; Interaction: genotype and treatment. (B,C) Primary hepatocytes from wild-type mice were cultured in control or Y/A/C-supplemented medium for 14 (B) or 10 days (C). B: Conditioned media (CM) or lysates were analyzed by western blot for VLK and TIMP2, or SOX9 and β-actin, where TIMP2 and β-actin were used as loading controls. Each lane represents an individual mouse. The blots are representative of three independent experiments. C: Immunofluorescence staining for VLK (red) combined with phalloidin-FITC staining to mark the actin cytoskeleton (green). Nuclei were counterstained with Hoechst 33342 (blue). *n*=3. (D) Representative images of primary hepatocytes from Ctrl or KO mice grown in 2D cultures in Y/A/C-supplemented medium for 11 or 14 days (left) and quantification of the area covered by cells (right). Dotted lines in the left panel indicate cell colony area. *n=*3 cultures from different mice per time point and genotype. (E) Representative images of primary hepatocytes from Ctrl or KO mice cultured in 3D in Y/A/C-supplemented medium over 14 days (left) and quantification (right) of colony diameter. Ctrl 1d: *n=*6, Ctrl 4d: *n=*3, Ctrl, 7d: *n=* 6, Ctrl 10d: *n=*6, Ctrl 14d: *n=*6, KO 1d: *n=*6, KO 4d: *n=* 3, KO 7d: *n=*6, KO 10d: *n=*6, KO 14d: *n=*6. Data are from two independent experiments. Arrowheads in the left panel indicate examples of how the colony diameter was measured. *P*-values are given for the following comparisons: diameter Ctrl Y/A/C cells and KO Y/A/C cells: day 7, *; day 9, #; day 14, °. (F) Representative images of hepatocytes from Ctrl or KO mice cultured in 3D in the presence of the indicated culture medium (left) and quantification of colony diameter (right) over 14 days. Ctrl 2d: *n=*4, Ctrl 5d: *n=*7, Ctrl 9d: *n=*7, Ctrl 12d: *n=*4, Ctrl 14d: *n=*4. Arrowheads in the left panel indicate examples of how the colony diameter was measured. *P*-values are given for the following comparisons: Condition, Y/A/C Ctrl or KO cells with Y/A/C medium, CM Ctrl or CM KO; Timepoint, 2, 5, 9, 12 and 14 d; Interaction: condition and timepoint. Data are from two independent experiments. (G) Representative images of immortalized mouse embryonic fibroblasts (MEFs) from wild-type mice incubated in the presence of the indicated culture medium over 10 days. Nuclei were counterstained with Hoechst 33342 (blue, top) and the Hoechst 33342-stained area was quantified (bottom). Three images per condition per mouse were quantified. Y/A/C-supplemented medium: *n=*8, CM Ctrl: *n=*8, CM KO: *n=*8. Result of three independent cell culture experiments is shown. Every data point represents conditioned medium from one mouse. (H) Representative photomicrographs (left) of primary hepatocytes from Ctrl or KO mice and MEFs from wild-type mice incubated in the presence of the indicated culture medium and analyzed by immunofluorescence staining for PDGFR-α (red) and CK19 (green). Nuclei were counterstained with Hoechst 33342. Dotted lines represent cell colony area of Y/A/C cells. Graph (right) shows quantification of average area of the ‘liver progenitor’ islets (CK19 positive). Result of three independent cell culture experiments is shown; each dot represents a culture from a different mouse. Ctrl cells with Y/A/C-supplemented medium: *n=*8; Ctrl cells with CM Ctrl: *n=*8; Ctrl cells with CM KO: *n=*8; KO cells with Y/A/C medium: *n=*8; KO cells with CM Ctrl: *n=*8; KO cells with CM KO: *n=*8. Mean islet area of Ctrl cells cultured in Y/A/C-supplemented medium was set to 1. Graphs show mean±s.d. *P*-values are given for the following comparisons: Genotype, Ctrl and KO Y/A/C cells grown with MEFs; Treatment, Y/A/C medium, CM Ctrl and CM KO; Interaction: genotype and treatment. Statistical significance was determined by two-way ANOVA (A,G), unpaired non-parametric Mann–Whitney test (E), or one-way ANOVA with Tukey's multiple comparisons test (F,H). Scale bars: C, 20 μm; D–H, 100 μm.

Hepatocytes and cholangiocytes have a high secretory activity, therefore, we speculated that VLK is secreted from these cells and modifies their secretome, which may affect other liver cells in a paracrine manner. To test this possibility, we generated hepatocyte-specific *Pkdcc* knockout mice by breeding mice with floxed *Pkdcc* alleles (Probst et al., 2013) with mice expressing Cre recombinase under control of the albumin promoter (Alb-Cre mice) (Postic et al., 1999). The latter allow expression of transgenes in hepatocytes with an onset during late prenatal development, and thus during the time when VLK expression is high in this cell type (Postic et al., 1999; Weisend et al., 2009). The mice with hepatocyte-specific loss of VLK [Alb-VLK; designated knockout (KO) mice in this article] and respective control (Ctrl) mice with floxed *Pkdcc* alleles, but lacking Cre, were first used to establish an *in vitro* model to study autocrine and paracrine effects of the hepatocyte-derived secretome on fibroblast behavior. Consistent with the *in vivo* data, primary hepatocytes from adult Ctrl mice only weakly expressed VLK, which was only detectable at the RNA level (Fig. 2A). However, their *in vitro* reprogramming into expandable bipotent cells, which express the progenitor marker sex-determining region Y (SRY)-related high mobility group (HMG)-box gene 9 (SOX9) (Fig. 2A,B), strongly promoted VLK expression and secretion (Fig. 2A–C). Immunoblotting specificity was confirmed by detection of VLK in HEK 293T cells transiently transfected with a *Pkdcc* expression vector (Fig. S1F). Reprogramming was achieved with a combination of small molecules, including the rho-associated, coiled-coiled containing protein kinase (ROCK) inhibitor Y27632, transforming growth factor (TGF)-β inhibitor A-83-01 and glycogen synthase kinase (GSK3) inhibitor CHIR99021 (collectively referred to as Y/A/C) (Katsuda et al., 2017). Additionally, we detected a unique VLK peptide in the conditioned medium by mass spectrometry (Fig. S1G).

Expression of SOX9 was not affected by the loss of VLK (Fig. S1H,I). Furthermore, culture of primary hepatocytes from Ctrl and KO mice in Y/A/C-supplemented medium over 30 days resulted in a comparable upregulation of *Sox9* expression, a similar decrease in albumin (*Alb*) expression and induction of *Pkdcc* expression in Ctrl cells (Fig. 2A). VLK has previously been shown to negatively regulate hedgehog signaling (Kim et al., 2020). However, loss of *Pkdcc* in reprogrammed primary hepatocytes did not alter the expression of a major hedgehog target gene, *Gli1* (Fig. S1J), suggesting that the hedgehog pathway is not affected in our *in vitro* system.

Ctrl and KO primary hepatocytes grown in Y/A/C-supplemented medium in a 2D monolayer showed a similar proliferation rate as assessed by analysis of colony area and 3-(4,5-dimethylthiazol-2-yl)-2,5-diphenyltetrazolium bromide (MTT) assay (Fig. 2D; Fig. S1K, two left panels). Interestingly, however, when grown as spheroids in matrigel, KO cells formed bigger colonies compared to their Ctrl counterparts (Fig. 2E). To determine whether the spheroid phenotype results from differences in the secretome, we cultured Ctrl and KO cells in the presence of their conditioned media (CM). Indeed, the spheroid size of Ctrl cells was higher when cultured in the presence of CM from KO versus CM from Ctrl cells. Vice versa, the spheroid size of KO cells was smaller when grown in CM from control cells versus CM from KO cells (Fig. 2F). Taken together, these results suggest that VLK suppresses proliferation of progenitor cells by altering their secretome.

To assess whether the secretome influences fibroblast proliferation, we supplemented mouse embryonic fibroblasts (MEFs) with either fresh Y/A/C-supplemented medium or CM derived from Ctrl or KO cultures. Fibroblast proliferation was generally increased in the presence of CM compared to non-conditioned Y/A/C-supplemented medium (Fig. 2G). Remarkably, this increase was much more pronounced in the presence of CM from KO versus CM from Ctrl cells (Fig. 2G). In an additional assay, Ctrl or KO cells were seeded together with MEFs in a monolayer. Co-cultures showed a similar overall cell viability and proliferation rate as assessed by the MTT assay (Fig. S1K, two right panels). Surprisingly, in these settings, addition of CM from KO cells, but not CM from Ctrl cells increased the area of cells which were CK19 positive, but negative for the fibroblast marker platelet-derived growth factor receptor alpha (PDGFR-α, also known as PDGFRA) (Fig. 2H), suggesting that it promotes proliferation of the progenitor cells. However, Ctrl or KO cells grown under the same conditions, but in the absence of MEFs, did not show a difference in the progenitor islet area (Fig. S1L). These results were reproduced with up to eight independent primary cell isolates from individual mice. The viability of primary de-differentiated hepatocytes grown in 2D or 3D cultures was confirmed by simultaneously evaluating viable and dead cells using fluorescein diacetate (FDA) and propidium iodide (PI) (Fig. S1M). Together, these results strongly suggest that VLK modifies the secretome of de-differentiated hepatocytes, thereby suppressing proliferation of liver progenitor cells and also of fibroblasts.

### Loss of VLK in hepatocytes causes perivascular fibrosis and cholestasis

**Fig. 3.**
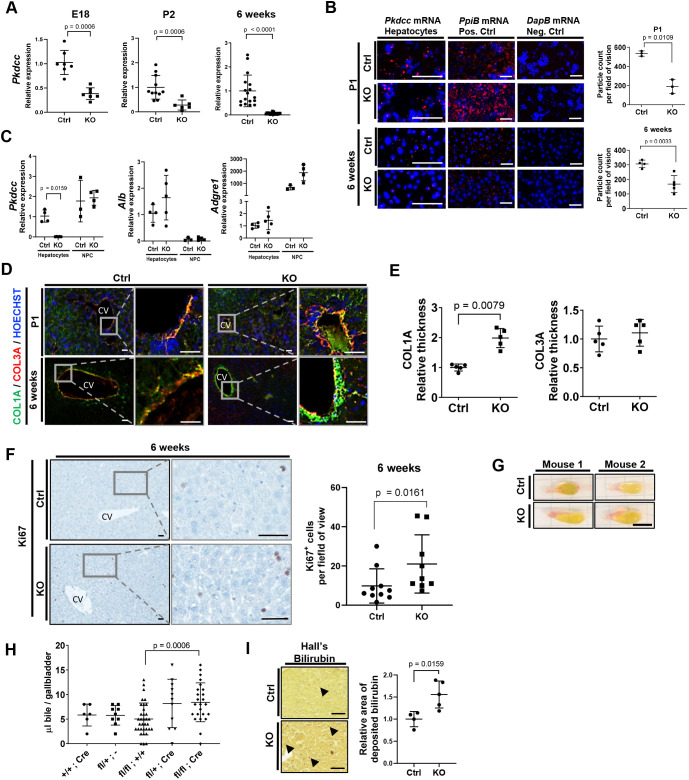
**Loss of VLK in hepatocytes promotes collagen deposition around vessels and cholestasis in adult mice.** (A) RNA from whole liver of Ctrl and KO mice at the indicated age was analyzed by qPCR for *Pkdcc* expression relative to *Rps18* expression*.* E18: *n*=7; P2: *n*=6–11; 6 weeks: *n=*17. Result of three independent RNA isolation experiments is shown. Mean expression levels in Ctrl mice were set to 1. (B) *Pkdcc* mRNA (red) in mouse liver at the indicated age, visualized by RNA-Scope *in situ* hybridization. *PpiB* and *DapB* mRNAs were used as positive and negative controls, respectively. Nuclei were counterstained with Hoechst 33342. P1: *n=*3; 6 weeks: *n=*4. Graphs (right) indicate particle count of positive-stained mRNA dots (red) per field of vision. (C) RNA from isolated primary hepatocytes and non-parenchymal cells (NPCs) of 6-week-old Ctrl and KO mice was analyzed by qPCR for *Pkdcc*, *Alb* and *Adgre1* expression relative to *Rps18* expression*.* Hepatocytes: *n*=4–5; NPC: *n*=3–4. Result of two independent isolation experiments is shown. Mean expression levels in hepatocytes of Ctrl mice were set to 1. (D) Representative photomicrographs of liver sections from Ctrl and KO mice at the indicated ages, analyzed by immunofluorescence staining for COL1 (green) and COL3A (red). Result of two independent experiments is shown (*n*=5 mice, *n*=3 images per mouse). Nuclei were counterstained with Hoechst 33342 (blue). CV, central vein. (E) Quantification of the perivascular area positive for each type of collagen in 6-week-old mice (*n=*5 mice; *n*=3 pictures per mouse). (F) Representative photomicrographs of liver sections analyzed by immunohistochemistry for anti-Ki67 (left) and quantification of Ki67^+^ cells in 6-week-old mice (right). *n=*9–10. Result of three independent tissue isolations is shown. CV, central vein. (G) Representative gallbladders isolated from 6-week-old Ctrl and KO mice. *n*=6. (H) Bile volume (μl) of gallbladders from 6-week-old Ctrl and KO mice. +/+;Cre: *n=*6; fl/+;−: *n=*9; fl/fl;+/+: *n=*34; fl/+;Cre: *n=*11; fl/fl;Cre: *n=*24. (I) Representative photomicrographs of liver sections from 6-week-old Ctrl and KO mice subjected to Hall's bilirubin staining (left) and quantification (right) of the area showing deposited bilirubin. Mean in Ctrl mice was set to 1. Arrowheads point to bilirubin crystals. *n*=4–5. Graphs show mean±s.d. Statistical significance was assessed by unpaired non-parametric Mann–Whitney test (A–C,E,F,I) or one-way ANOVA, followed by Tukey's multiple comparison test (H). Scale bars: B,D,F,I, 100 μm; G, 4 mm.

To unravel the role of VLK in hepatocytes for late perinatal and postnatal liver development and homeostasis, we analyzed the mice with the hepatocyte-specific VLK loss (KO). These animals differed neither in body or liver mass nor in the liver-to-body mass ratio from control littermates (Fig. S2A). The number of VLK-positive, clustered hepatocytes in E18 and particularly in P2 KO mice was significantly reduced (Fig. S2B), which is consistent with the onset of the activity of the albumin promoter in hepatocytes during late embryonic development (Postic et al., 1999; Weisend et al., 2009). *Pkdcc* expression in total liver tissue was strongly reduced in KO mice at E18 and P2 and almost undetectable in adult mice (Fig. 3A). This is expected since hepatocytes are by far the most abundant cell type in the liver (Stanger, 2015). RNA-Scope *in situ* hybridization confirmed the decrease in *Pkdcc* expression in hepatocytes during postnatal liver development (Fig. 3B). qPCR analyses of primary liver cells from 6-week-old mice, which had been fractionated into hepatocytes and non-parenchymal cells (NPCs), confirmed the hepatocyte-specific *Pkdcc* deletion (Fig. 3C). The purity of the fractions was verified by expression analysis of the hepatocyte-specific marker *Alb* and the NPC-specific marker adhesion G-protein-coupled receptor 1 (*Adgre1*) (Fig. 3C). VLK expression in cholangiocytes was not affected in the KO mice (Fig. S2C). This was confirmed by the absence of tandem-dimer-Tomato (tdTomato) expression in bile ducts of mice expressing the fluorescent reporter in a Cre-dependent manner (tdTomato reporter mice) (Fig. S2D)*.* Loss of VLK in hepatocytes affected neither the expression of the liver progenitor marker *Sox9* or the cholangiocyte marker secreted phosphoprotein 1 (*Spp1*) (encoding osteopontin, OPN), nor the localization of these proteins in the developing and adult liver (Fig. S2E,F). Furthermore, immunofluorescence detection of bile canaliculi (positive for cluster of differentiation 26, CD26; also known as DPP4) and blood vessels (positive for pan-endothelial cell antigen, MECA-32; also known as PLVAP) did not reveal major differences between KO and Ctrl littermates (Fig. S2F).

Because of the previously suggested role of VLK in matrix remodeling (Bordoli et al., 2014) and the strong effect of the CM of de-differentiated VLK KO hepatocytes on fibroblasts, we evaluated the expression of collagen I and collagen III (COL1A1 and COL3A1 subunits, respectively) by immunofluorescence staining. There was no obvious difference in collagen deposition in new-born mice, but collagen I strongly accumulated in adult KO mice, in particular around blood vessels and most prominently around the central vein (Fig. 3D,E), whereas *Col1a1* and *Col3a1* gene expression in whole liver tissue was not affected (Fig. S2G). Proliferation assessed by Kiel-Antigen No. 67 (Ki67, also known as MKI67) staining revealed more proliferative cells in adult KO mice compared to those in Ctrl mice (Fig. 3F), but no changes between Ctrl and KO mice at E18 or P1 (Fig. S2H).

During necropsy, we noticed an enlarged gallbladder in adult KO mice (Fig. 3G). This is most likely the result of an increase in the amount of stored bile liquid (Fig. 3H; Fig. S2I). Moreover, there was a significant increase in deposited bilirubin crystals in the livers of KO mice (Fig. 3I), suggesting impaired bile flow. The fibroblast growth factor 15 (Fgf15)/Fgf receptor 4 (Fgfr4) pathway inhibits bile acid synthesis and accumulation of toxic bile acids in the liver by blocking the expression of cholesterol 7 α-hydroxylase (*Cyp7a1*) and stimulation of gallbladder filling (Inagaki et al., 2005). However, neither *Cyp7a1* nor *Fgfr4* expression was altered in the liver of 6-week-old (Fig. S3A) or 1-year-old (Fig. S3B) KO mice, suggesting that alterations in this pathway are not responsible for the bile phenotype. Despite these abnormalities, there was no major liver damage as suggested by the normal serum levels of aspartate aminotransferase (AST), alanine aminotransferase (ALT) and alkaline phosphatase (ALP) (Fig. S3C,D). Since bile acid composition can influence bile flow over time (Söderdahl et al., 1998; Wannagat et al., 1978; Li et al., 2017), we evaluated the bile acid composition from the livers and bile of KO and Ctrl mice (Fig. S3E–H). In 6-week-old mouse livers, the overall bile acid composition was not affected by the knockout (Fig. S3E), whereas in 1-year-old mouse livers, the total bile acids, the taurine-conjugated bile acids and the primary bile acids were increased in the liver tissue of the mutant mice (Fig. S3F). However, the overall bile acid composition in the gallbladder did not change at either time point (Fig. S3G,H).

Because VLK expression is particularly strong in cholangiocytes of adult mice (Fig. 1B), we also determined whether an acute loss of VLK in the biliary epithelium affects liver homeostasis. To address this question, we crossed mice with floxed *Pkdcc* alleles with transgenic mice expressing a tamoxifen-inducible Cre recombinase [Cre^(ERT)^] under control of the cytokeratin 19 (CK19) promoter [CK19-Cre^(ERT)^]. In parallel, CK19-Cre^(ERT)^ mice were also crossed with a tdTomato reporter strain to determine potential leakiness of the inducible system and the cell type specificity of the Cre-mediated recombination (Means et al., 2008). In the absence of tamoxifen, some tdTomato fluorescence was observed in very few cells of the bile duct and lung bronchi (Fig. S3I, left panel). Upon tamoxifen treatment, however, strong and specific tdTomato expression was seen in cholangiocytes and in known CK19-positive bronchial cells (Fig. S3I, right panel). Neither the amount of bile nor the body mass were affected by the knockout (Fig. S3J). Co-immunofluorescence staining for OPN or CK19 with VLK confirmed the loss of VLK in cholangiocytes (Fig. S3K,L). Neither liver morphology nor collagen deposition around the blood vessels was affected by the knockout as determined by Herovici staining (Fig. S3M). Therefore, we focused our next experiments on mice lacking VLK in hepatocytes.

### Loss of VLK in hepatocytes causes liver steatosis and inflammation in aged mice

**Fig. 4.**
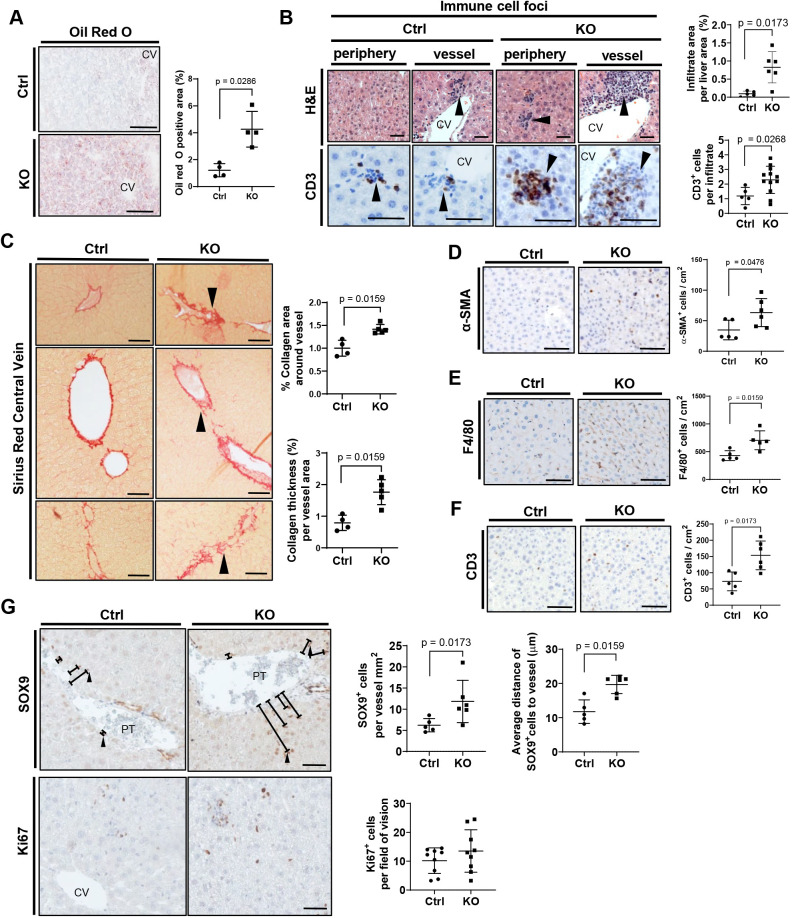
**Loss of hepatocyte-derived VLK causes a steatohepatitis-like phenotype in aged mice.** (A) Representative photomicrographs (left) and quantification (right) of Oil Red O staining of liver sections from Ctrl or KO mice. CV, central vein. *n*=4 mice, *n*=3 pictures per mouse. (B) Representative photomicrographs of liver sections stained with Hematoxylin and Eosin (H&E) or analyzed by CD3 immunohistochemistry (left), and percentage of the liver area covered by immune cells or CD3^+^ cells (right). Arrowheads in the left panel indicate immune cell foci. CV, central vein. Ctrl: *n*=5, KO *n*=6 (immune cells based on H&E staining); Ctrl: *n=*5, KO: *n=*11 (CD3^+^ cells). Mean in Ctrl mice was set to 1. (C) Representative photomicrographs of liver sections focused on vein structures and stained with Sirius Red (left), and quantification of the Sirius Red-positive area around vessels and average collagen thickness per vessel area (right). Arrowheads in the left panel indicate increased fibrotic areas. *n=*5–6 mice, *n*=3 pictures per mouse. (D–F) Representative photomicrographs of liver sections analyzed by immunohistochemistry for α-SMA (D), F4/80 (E) or CD3 (F) (left) and quantification of α-SMA-, F4/80- or CD3- positive cells (right). *n=*3–6 mice, *n*=3 pictures per mouse. Mean in Ctrl mice was set to 1. (G) Representative photomicrographs of liver sections analyzed by immunohistochemistry for SOX9 and Ki67 (left) and quantification of SOX9^+^ cells per vessel area and of average distance of SOX9^+^ cells from vessels, as well as quantification of Ki67^+^cells per live area (right). Arrowheads in the left panel indicate examples of measured distance from the vessel wall to the SOX9^+^ cell. PT, portal triad; CV, central vein. *n=*5–9 mice, *n*=3 pictures per mouse. Graphs show mean±s.d. Statistical significance for all panels in this figure was assessed by unpaired non-parametric Mann–Whitney test. Scale bars: 100 μm.

The increase in bile acids in the livers of 1-year-old mice that lack VLK in hepatocytes suggested that the phenotype may aggravate upon aging. Therefore, we further analyzed the mutant mice at 1 year of age. The efficiency of the *Pkdcc* knockout was confirmed at this time point (Fig. S4A,B). Although body and liver mass were still unaffected (Fig. S4C), steatosis with signs of inflammation and fibrosis developed in KO mice, characterized by increased numbers of lipid droplets (Fig. 4A), accumulation of cluster of differentiation 3 (CD3)-positive T cells around vessels and in the periphery of the liver lobules (Fig. 4B) and more pronounced perivascular collagen deposition (Fig. 4C). In addition, the numbers of alpha smooth muscle actin (α-SMA)-positive (activated) stellate cells and of cell surface glycoprotein F4/80 (F4/80)-positive macrophages were significantly increased in the liver of 1-year-old KO mice (Fig. 4D–F), although there was no obvious increase in α-SMA-positive cells around the vessels or in B cell isoform 220 kDa (B220)-positive cells, which represent B cells (Fig. S4D,E). Expression of the pro-inflammatory cytokines interleukin (IL)-1β, IL-6 and tumor necrosis factor α (*Il1b*, *Il6* and *Tnfa*) showed no significant change, but exhibited a trend towards higher mRNA levels in the KO mice (Fig. S4F). Most interestingly, SOX9-positive cells, which most likely represent progenitor cells, were more abundant and were found at a further distance from the portal area in KO livers (Fig. 4G, upper panels). However, the overall liver cell proliferation did not change (Fig. 4G, lower panels), and there were no signs of tumor formation. These findings suggest increased motility and recruitment of progenitor cells in the absence of hepatocyte-derived VLK (e.g. ductular reactions), although activation and expansion of cholangiocytes cannot be excluded.

In line with the *in vitro* data (Fig. S1J), the expression of the classical hedgehog target genes *Gli1* and *Ccnd1* was not altered in the liver tissue of either young or aged Ctrl and KO mice (Fig. S4G) or in fractionated hepatocytes and NPCs (Fig. S4H) of Ctrl and KO mice. Additionally, in agreement with data from younger mice (Fig. S3M), aged mice with the CK19-Cre^(ERT)^-mediated knockout did not show obvious differences in collagen deposition or overall liver morphology as assessed by Sirius Red staining (Fig. S4I).

### VLK in hepatocytes affects the pattern of deposited collagen after chronic liver injury

**Fig. 5.**
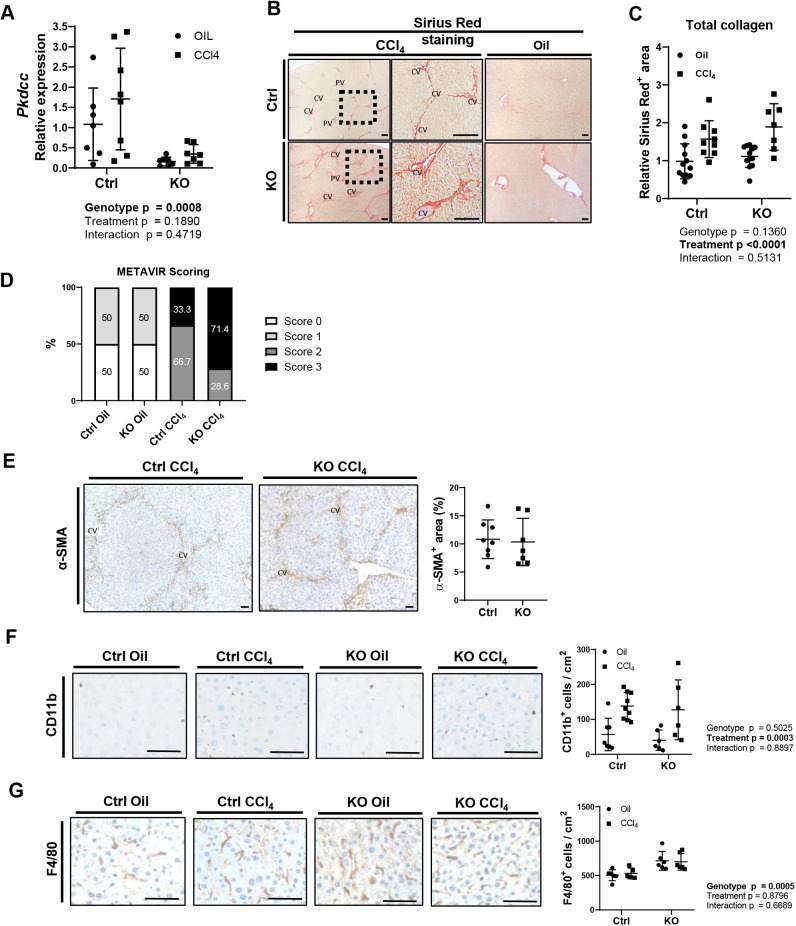
**Loss of hepatocyte-derived VLK alters the pattern of deposited collagen after chronic CCl_4_ treatment.** (A) RNA from the liver of Ctrl and KO mice chronically treated with CCl_4_ or vehicle (oil) was analyzed for *Pkdcc* expression relative to *Rps18* expression by qPCR. Ctrl oil: *n=*7; Ctrl CCl_4_: *n=*9; KO oil: *n=*7; KO CCl_4_: *n=*7. Mean expression levels in Ctrl mice were set to 1. *P*-values are given for the following comparisons: Genotype, control and KO; Treatment, vehicle oil and CCl_4_; Interaction: genotype and treatment. (B) Representative photomicrographs of Sirius Red-stained liver sections from Ctrl or KO mice following chronic oil or CCl_4_ treatment. Ctrl oil: *n=*11; Ctrl CCl_4_: *n=*9; KO oil: *n=*10; KO CCl_4_: *n=*7. PV, portal vein; CV, central vein. (C) Quantification of Sirius Red-stained area in liver sections from Ctrl or KO mice following chronic oil or CCl_4_ treatment. Ctrl oil: *n=*11; Ctrl CCl_4_: *n=*9; KO oil: *n=*10; KO CCl_4_: *n=*7. Mean in Ctrl mice was set to 1. *P*-values are given for the following comparisons: Genotype, control and KO; Treatment, vehicle oil and CCl_4_; Interaction: genotype and treatment. (D) Sirius Red-stained liver sections were evaluated by the semi-quantitative METAVIR scoring system to stage liver fibrosis. METAVIR scoring adapted to discriminate between bridging fibrosis with few septae (Score 0) vs many septae (Score 3). Ctrl Oil: *n=*6; KO Oil: *n=*6; Ctrl CCl_4_: *n=*9; KO CCl_4_: *n=*7. (E) Representative liver sections from Ctrl and KO mice chronically treated with CCl_4_ or vehicle (oil), analyzed by immunohistochemistry for α-SMA and co-staining with Hematoxylin and Eosin (H&E) (left), and quantification of the α-SMA^+^ area (right). Ctrl CCl_4_: *n=*8; KO CCl_4_: *n=*7. CV, central vein. (F,G) Representative liver sections from Ctrl and KO mice chronically treated with CCl_4_ or vehicle (oil), analyzed by immunohistochemistry for CD11b (F) or F4/80 (G), and co-stained with hematoxylin (left); quantification of the CD11b^+^ and F4/80^+^ cells per area (right). Ctrl oil: *n=*6–7; Ctrl CCl_4_: *n=*5–9; KO oil: *n=*6; KO CCl_4_: *n=*6. Data from one out of two independent experiments are shown. Graphs show mean±s.d. *P*-values are given for the following comparisons: Genotype, control and KO; Treatment, vehicle oil and CCl_4_; Interaction: genotype and treatment. Statistical analysis was performed using two-way ANOVA (A,C,F,G) or unpaired non-parametric Mann–Whitney test (E). Scale bars: 100 μm.

The findings described above suggest a protective function of VLK in hepatocytes under homeostatic conditions, particularly in perivascular areas. Therefore, we next determined whether chronic toxic liver injury in response to repeated treatment with CCl_4_ (Fig. S5A) and the resulting fibrosis is aggravated in KO mice. Chronic CCl_4_ treatment mildly, but not significantly, increased *Pkdcc* expression in both Ctrl and KO mice (Fig. 5A). The observed increase in the KO mice is most likely due to *Pkdcc* expression in non-parenchymal cells. The liver to body mass ratio was neither affected by CCl_4_ treatment nor by genotype (Fig. S5B). Loss of VLK also had no effect on the total number of proliferating (Ki67-positive), apoptotic (cleaved caspase-3-positive) (Fig. S5C) or SOX9-positive cells in mice treated with CCl_4_ or vehicle (oil) (Fig. S5D). As expected, *Col1a1* and *Col3*a*1* expression, as well as Sirius Red-positive areas increased in the CCl_4_-treated mice, whereas the overall Sirius Red-positive area did not differ between genotypes (Fig. S5E; Fig. 5C). However, the pattern of collagen deposition was altered in KO mice (Fig. 5B). Whereas thin, regular collagen filaments bridged the vessels in Ctrl mice, more extensive, patchy collagen deposition was detected in KO mice, particularly around blood vessels. The METAVIR scoring used to determine the degree of fibrosis by discriminating between bridging fibrosis with few versus many septae (Bedossa and Poynard, 1996) showed a clear trend towards more severe fibrosis (score 3) in KO animals treated with CCl_4_ (Fig. 5D).

This fibrotic pattern was also observed by staining for the (myo)fibroblast marker α-SMA (Fig. 5E). Consistent with a lack of global increase in fibrosis, the α-SMA-positive liver area as well as expression of actin alpha-2 (*Acta2)* (encoding α-SMA) in whole liver tissue were not affected by the loss of VLK (Fig. 5E; Fig. S5F). *Pdgfra* expression increased following CCl_4_ treatment, and this was slightly more pronounced in KO mice (Fig. S5F). There were no significant differences in cluster of differentiation 11 antigen-like family member B (CD11b)^+^ myeloid cells or in B220^+^ B cells, neither upon oil nor upon CCl_4_ treatment (Fig. 5F; Fig. S5G). However, the number of liver-resident F4/80^+^ macrophages was increased in KO livers, although their numbers were not affected by CCl_4_ treatment (Fig. 5G). CD3^+^ T cells were more abundant in oil-treated KO versus Ctrl mice, whereas no difference was seen in CCl_4_-treated mice (Fig. S5G). In contrast to mice lacking VLK in hepatocytes, CK19-Cre^(ERT)^-mediated *Pkdcc* KO mice did not show an obvious phenotype upon chronic CCl_4_ injury as determined by Sirius Red staining (Fig. S5H). Together, these results suggest that hepatocyte-derived VLK affects the pattern rather than the extent of collagen deposition in the liver upon chemically induced fibrosis and only mildly affects the immune cell response. These *in vivo* abnormalities may result from alterations of the hepatocyte secretome as shown in our *in vitro* studies, which locally affects adjacent stromal cells including fibroblasts.

### Loss of VLK in hepatocytes promotes AFP secretion

**Fig. 6.**
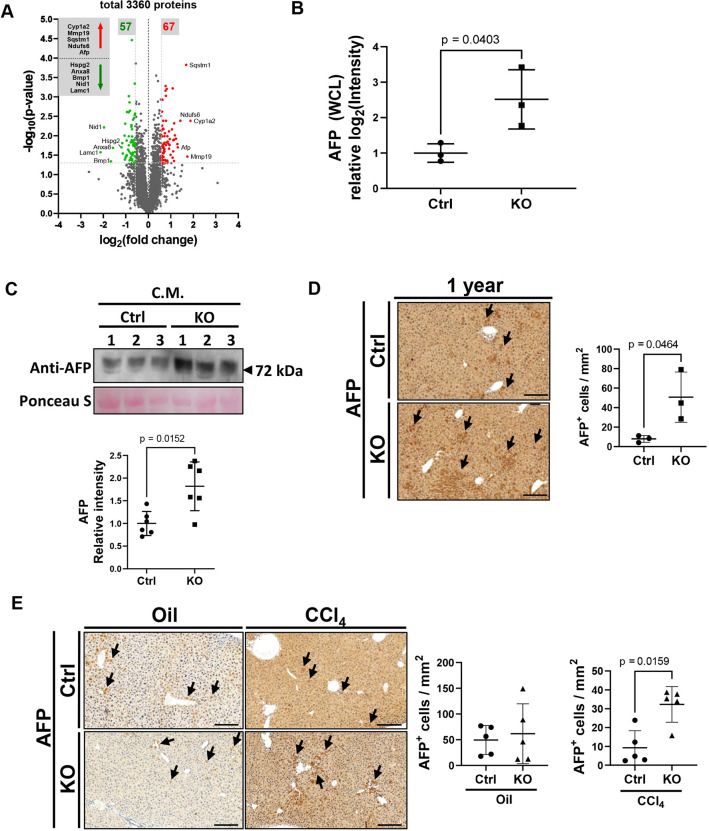
**Loss of hepatocyte-derived VLK promotes AFP secretion.** (A) Volcano plot of all proteins detected with LC-MS/MS in whole cell lysates (WCL) of Y/A/C-treated hepatocytes [log_2_(fold change)>0.58; −log_10_(*P*-value)>1.3; paired two-tailed *t*-test, *P*<0.05, *P*-values were not corrected for multiple testing]. Proteins with significantly higher abundance in KO cells are labeled in red, those with significantly lower abundance in KO cells are labeled in green. Ctrl: *n=*3; KO: *n=*3. (B) Mass spectrometry relative log_2_(intensity) of measured AFP in the WCL of Y/A/C-treated cells of both genotypes. Ctrl: *n=*3, KO; *n=*3. (C) Conditioned media (CM) of Y/A/C-treated hepatocytes were analyzed for AFP by western blotting. Representative western blot for AFP (top) and quantification of AFP intensity relative to Ponceau S staining (bottom, two western blots from two independent experiments) are shown. Ponceau S was used to verify equal loading. Ctrl: *n=*6; KO: *n=*6. (D) Representative immunohistochemistry images (left) and quantification (right) for AFP of 1-year-old Ctrl or KO livers. Sections were counterstained with hematoxylin. Ctrl: *n=*3; KO: *n=*3. Arrows in the left panel indicate AFP^+^ cells. (E) Representative immunohistochemistry images (left) and quantification (right) for AFP of Ctrl or KO livers chronically treated with CCl_4_ or vehicle (oil) (left). Sections were counterstained with hematoxylin. Ctrl Oil: *n=*6, KO Oil: *n=*6, Ctrl CCl_4_: *n=*6, KO CCl_4_: *n=*6. Arrows in the left panel indicate AFP^+^ cells. Graphs show mean±s.d. Statistical significance was assessed by unpaired two-tailed *t*-test (A) or unpaired non-parametric Mann–Whitney test (B–E). Scale bars: 200 μm.

Our study suggests expansion of progenitor cells in the liver of aged VLK KO mice and supports a role for secreted factors in modulating the proliferation of fibroblasts in a VLK-dependent manner. To identify proteins that are differentially abundant in reprogrammed hepatocytes from Ctrl and VLK KO mice, we performed a proteomic analysis of whole cell lysates from three Ctrl and three KO mice. Out of 3360 identified proteins, 57 were significantly less abundant and 67 were significantly more abundant in the lysates of VLK KO reprogrammed cells (Fig. 6A; paired two-tailed *t*-test, *P*<0.05). Principal component analysis (PCA) of Ctrl and KO lysates showed a clear clustering (Fig. S6A). Since our reported phenotypes are most likely mediated by secreted factors, we focused on the significantly more or less abundant proteins with reported signal peptides (Fig. S6B). Among them, alpha-fetoprotein (AFP), a known marker for liver injury and hepatocellular carcinoma secreted by hepatic progenitor cells (Alison et al., 2009) was significantly more abundant in the lysates of reprogrammed VLK KO cells (Fig. 6B). Importantly, western blot analyses showed that AFP protein levels are also increased in the secretome of reprogrammed VLK-deficient hepatocytes in culture (Fig. 6C; Fig. S6C). AFP mRNA levels neither differed in liver tissue nor in reprogrammed hepatocytes from Ctrl versus KO mice (Fig. S6D), suggesting regulation at the protein level. In line with the *in vitro* findings, immunohistochemical staining showed a significant increase in cells with strong AFP staining in tissue sections of 1-year-old *Pkdcc* KO mice (Fig. 6D). Furthermore, AFP-positive cells were also significantly increased in the liver of the mutant mice upon chronic CCl_4_ injury (Fig. 6E). Taken together, these data suggest that VLK-dependent modulation of AFP may at least in part be responsible for the reported *in vitro* and *in vivo* phenotypes.

## DISCUSSION

We identified a protective function of VLK in the liver under homeostatic conditions and in response to toxin-induced injury. This effect is mediated via hepatocyte-derived VLK as shown by the perivascular fibrosis in mice lacking *Pkdcc* in this cell type. By contrast, inducible knockout of *Pkdcc* in cholangiocytes had no obvious effect. It should be considered, however, that the knockout of *Pkdcc* in hepatocytes already occurred during late embryonic development, whereas the knockout in cholangiocytes was induced in adult mice. In the future it will therefore be interesting to induce the knockout in cholangiocytes at an earlier stage.

The most obvious phenotype in young mice with hepatocyte-specific *Pkdcc* knockout was the progressive perivascular fibrosis, in particular around the central vein. In addition, we observed mild cholestasis characterized by higher bile volumes in the gallbladder and increased deposition of bilirubin throughout the liver. However, markers of liver injury or liver function in the blood were not altered, suggesting that any damage to the liver is rather localized. It may well be that alterations in the secretome of VLK-deficient hepatocytes predominantly affect cells around vessels, including pericytes and vascular smooth muscle cells. This could result in inappropriate collagen deposition and its organization into an abnormal 3D network. Moreover, the increased bile production combined with the effect of VLK deficiency on ductular cells may cause dysregulation of bile transport. This could result in tissue damage and inflammation because of the strong detergent properties of bile acids. Consistent with this possibility, aged KO mice developed signs of steatosis, combined with mild inflammation and fibrosis.

Surprisingly, loss of VLK in hepatocytes mainly altered the fibrotic pattern upon chronic CCl_4_ injury, whereas the overall extent of collagen deposition was not affected. These findings suggest that VLK is not a general anti-fibrotic protein, not even in response to an insult. Rather, it may regulate collagen deposition through a yet unknown direct or indirect mechanism, which could also involve other matrix proteins. Interestingly, VLK-dependent phosphorylation in close proximity to the C-terminal cleavage site in COL1A1 was previously reported, which might affect its processing (Bordoli et al., 2014). Therefore, altered collagen processing in *Pkdcc* knockout mice could contribute to the abnormal pattern of collagen deposition. Although NPCs also express *Pkdcc* (Fig. 3C), the loss of hepatocyte-derived VLK cannot be entirely compensated by VLK that is released from other cell types. This may be a consequence of limited diffusiveness and/or VLK activity in the endoplasmic reticulum or Golgi complex of hepatocytes as previously shown for other cell types (Bordoli et al., 2014). Therefore, it will be of interest in the future to determine the role of VLK in NPCs of the liver, including stellate cells. Although an intracellular activity of VLK may also be relevant in hepatocytes, effect of VLK deficiency in this cell type on collagen deposition in the liver indicates that VLK acts at least in part in a paracrine manner. This hypothesis is strongly supported by our *in vitro* studies with Y/A/C-treated hepatocytes. In contrast to freshly isolated primary hepatocytes, these de-differentiated cells robustly express and secrete VLK. Together with the strong expression of VLK in hepatocytes of neonatal mice and its downregulation in fully differentiated hepatocytes of adult mice and concomitant upregulation in bile ducts, this finding suggests that high VLK activity characterizes cells with a progenitor-like state. This hypothesis is in line with the induction of terminal cellular differentiation in the liver parenchyme after birth (Cannon et al., 2016). Our *in vitro* studies further suggest that VLK expression and/or secretion are functionally relevant for progenitor cells, since the secretome of VLK-deficient cells promoted their migration and proliferation. Therefore, VLK may be required for the limitation of progenitor cell proliferation *in vitro* and possibly also *in vivo* as suggested by the increase in SOX9- and CK19-positive progenitor cells around large liver vessels in *Pkdcc* KO mice and their abnormal distribution. In the future, it will be interesting to determine the consequences of VLK loss in hepatocytes for liver repair under conditions that induce activation of progenitor cells or expansion of cholangiocytes, such as the 3,5-diethoxycarbonyl-1,4-dihydrocollidine (DDC) diet (Pose et al., 2019). Interestingly, proliferation of progenitor cells *in vitro* was only affected in cells grown in spheroids or 2D co-cultures with mesenchymal cells. The latter finding suggests that VLK in the hepatocyte supernatant affects fibroblasts, which in turn produce factors that regulate hepatocytes. Alternatively, VLK may phosphorylate proteins that are released by fibroblasts. A role for fibroblasts in supporting hepatocyte metabolism and physiology is consistent with previous data and constitutes the basis of micropatterned co-cultures, a 2D system used to assess liver toxicity in drug screening programs (Khetani and Bhatia, 2008; March et al., 2015).

Identification of the proteins, which are affected by VLK-deficiency in the hepatocyte secretome and responsible for the phenotype in the KO mice will be a major goal of future studies. In a first attempt to identify these proteins, we performed quantitative proteomic analyses of cultured VLK-deficient and control progenitor cells. AFP was among the few differentially abundant proteins which also have a signal peptide. Importantly, it was also present at higher levels in the conditioned medium of VLK-deficient Y/A/C-treated cells. AFP is a clinically relevant marker of liver injury (Alison et al., 2009) and its upregulation is in line with the phenotype observed in aged *Pkdcc* KO mice. Interestingly, AFP has been shown to increase the proliferation rate of fibroblasts (Li et al., 2002). Its upregulation in cells from KO mice therefore provides a potential explanation for their positive effect on fibroblast proliferation. Follow-up *in vitro* studies should address the VLK-dependent regulation of AFP expression and/or secretion and determine whether AFP is a direct phosphorylated substrate of VLK or if its increased abundance is a consequence of the de-differentiation of hepatocytes to progenitor-like cells in the absence of VLK.

In summary, our data strongly suggest a role for VLK in the regulation of progenitor cells *in vitro* and *in vivo* and a hepato-protective function for VLK, which is particularly relevant during aging and in response to chronic liver injury. These results may well be relevant for human liver disease. Thus, the effect of VLK on the bile acid pool and bile homeostasis, and the development of pericentral steatosis and liver inflammation in the *Pkdcc* knockout mice raises the interesting question if impaired VLK expression plays a role in the pathogenesis of steatohepatitis. In the future, it will be interesting to determine whether particular diets (i.e. high fat, high sucrose or western diets) further increase the susceptibility to steatosis and progression to steatohepatitis in mice lacking VLK in hepatocytes, and if expression or activity of VLK is downregulated in distinct subsets of hepatocytes of patients with hepatic steatosis and/or fibrosis. This will reveal if modulation of VLK activity or targeting its substrates can be explored for therapeutic purposes.

## MATERIALS AND METHODS

### Mice

Mice were maintained under Specific Pathogen Free (SPF) conditions at the ETH Zurich Phenomics Center (EPIC) and received food and water *ad libitum*. All experiments involving animals had been approved by the responsible veterinary authorities (Cantonal Veterinary Office Zurich). Mice with floxed *Pkdcc* alleles (Probst et al., 2013) [designated control (Ctrl) mice] were mated with mice expressing Cre recombinase under control of the albumin promoter (Alb-Cre mice) (Postic et al., 1999) to generate mice with hepatocyte-specific loss of VLK [Alb-VLK; designated knockout (KO)]. In addition, we used tdTomato reporter mice [Ai14(RCL-tdT)-D, #007914, Jackson Laboratory, Bar Harbor, MA]. All mice were in C57BL/6 genetic background. Mice of mixed gender were used in all the experiments. The exact sample size and age of mice used in each experiment is indicated in the figure legends. Additionally, mice with the floxed *Pkdcc* alleles (Probst et al., 2013) were crossed with a tamoxifen-inducible version of Cre recombinase under the control of the cytokeratin 19 promoter [*CK19-*Cre^(ERT)^ mice] (Means et al., 2008).

### Genotyping

Mouse genotyping was performed by PCR analysis of DNA, which had been isolated from ear biopsies using the KAPA2G FAST Genotyping Mix (2GFKB, Roche, Rotkreuz, Switzerland). Amplified PCR products were visualized by gel electrophoresis. Details of the genotyping primers can be found in Table S1.

### Tissue and cell processing

Tissue samples were isolated and fixed overnight at 4°C in 4% paraformaldehyde (PFA) (#P6148, Sigma-Aldrich, St Louis, MO) or acetic ethanol (25% acetic acid glacial, 75% ethanol), embedded in paraffin and sectioned (3 µm thickness). Alternatively, unfixed tissue was immediately frozen in Tissue Freezing Medium^®^ (#14020108926, Leica Biosystems, Wetzlar, Germany) and sectioned (5 µm). Cultured cells were fixed in 4% PFA for 30 min at room temperature.

### Hematoxylin and Eosin staining

For Hematoxylin and Eosin (H&E) staining, paraffin sections were deparaffinized, rehydrated using a xylene/ethanol gradient, stained with Hematoxylin (#M906-03, JT Baker^®^, Phillipsburg, NJ) and Eosin-Y alcoholic (#102439, Merck, Darmstadt, Germany) and mounted with Eukitt^®^ (#03989, Sigma-Aldrich).

### Hall's bilirubin staining

Frozen sections were stained as previously described (Hall, 1960) and mounted with Eukitt^®^ (#03989, Sigma-Aldrich).

### Oil Red O staining

Oil Red O staining was performed as previously described (Wilson, 1950). Briefly, cryosections were fixed in 4% PFA for 10 min, washed in PBS and incubated twice for 5 min in 85% propylene glycol (#398039-500ML, Sigma-Aldrich). They were then incubated in Oil Red O solution for 7 min [0.7 mg Oil Red O (#O-0625, Sigma-Aldrich) in 100 ml propylene glycol, heated to 60°C]. Nuclei were counterstained for 2 min with H&E, and slides were mounted with Mowiol^®^ 4-88 (#81381, Sigma-Aldrich).

### Herovici staining

Herovici staining was performed to assess collagen deposition in a tissue and to distinguish between young and mature collagen. Herovici is a polychromatic staining (young collagen is blue, mature collagen appears pink to red, cytoplasm stains in yellow). The staining was performed according to previously described protocols (Herovici, 1963; Teuscher et al., 2019).

### Sirius Red staining

Formalin-fixed and paraffin embedded (FFPE) sections were deparaffinized and rehydrated using a xylene/ethanol gradient. Tissues were stained as previously described (Junquiera et al., 1979) using Sirius Red (Direct Red 80, #43665, Sigma-Aldrich).

### Immunohistochemistry and immunofluorescence staining

For immunohistochemistry (IHC) and immunofluorescence (IF) staining, FFPE- and acetic-ethanol-fixed sections were dewaxed and rehydrated using a xylene/ethanol gradient. For FFPE sections we added an antigen retrieval step by incubation in citrate buffer (10 mM citric acid, pH 6) at 95°C for 1 h, followed by several washes with PBS supplemented with Tween-20 (PBS-T; 137 mM NaCl, 2.7 mM KCl, 10 mM Na_2_HPO_4_, 2 mM KH_2_HPO_4_, 0.1% Tween-20). All samples were blocked with 12% bovine serum albumin (BSA) in PBS for 1 h at room temperature, followed by incubation with the primary antibody overnight at 4°C and for 2 h with the secondary antibody at room temperature. Hoechst 33342 was used to counterstain nuclei. For IHC detection we used the Vectastain^®^ ABC-horse radish peroxidase (HRP) Kit (#PK4000, Vector Laboratories, Burlingame, CA) and the DAB Substrate Kit (#SK-4100, Vector Laboratories). The following antibodies were used for immunostaining: rat anti-cytokeratin 19 (CK19) (1:500, TROMA-III, Developmental Studies Hybridoma Biobank DSHB, Iowa City, IO), rabbit anti-VLK (1:300, #TA306903, Origene, Rockville, MD), rabbit anti-VLK 289 and rabbit anti-VLK 404 (1:300, Whitman laboratory, Harvard University, Boston, MA), goat anti-osteopontin (1:300, #AF808, R&D Systems, Minneapolis, MN), goat anti-PDGFR-α (1:400, #AF1062, R&D Systems), goat anti-Alb (1:100, #A80-129A, Bethyl Laboratories, Montgomery, TX), rabbit anti-AFP (1:300, #14550-1-AP, Proteintech, Rosemont, IL), rabbit anti-SOX9 (1:500, #AB5535, Merck Millipore, Burlington, MA), rat anti-Meca-32 (1:500, #553849, BD Pharmingen, San Diego, CA), rat anti-CD31 (1:500, #553370, BD Pharmingen), goat anti-collagen1 (1:300, #1310-01, Southern Biotech, Birmingham, AL), rabbit anti-collagen 3 (1:300, #Ab7778, Abcam, Cambridge, UK), rabbit anti-Ki67 (1:500, #ab15580, Abcam), goat anti-DPPIV/CD26 (1:400, #AF954, R&D Systems), rabbit anti-cleaved caspase 3 (1:400, #9661, Cell Signaling), multilabeling donkey anti-rabbit Cy3 (1:500, #711-165-152, Jackson ImmunoResearch, Westgrove, PA), multilabeling donkey anti-goat Cy2 (1:500, 705-225-147, Jackson), Alexa Fluor 488 donkey anti-rat (1:500, A21208, Invitrogen, Carlsbad, CA), and anti-rabbit IgG HRP Conjugate (1:500, pH 6, W4011, Promega, Madison, WI). In addition, we used Phalloidin-FITC (1:1000, P5282, Sigma-Aldrich) to stain the actin cytoskeleton.

For IHC staining of murine lymphoid cells (CD3, CD11b, F4/80, and B220) and for α-SMA and collagen IV staining, slides were stained on two different platforms: Ventana Benchmark Discovery (Ventana Medical Systems, Inc, Tucson, AZ) and Bond (Leica Biosystems) according to the manufacturers’ protocols. Primary and secondary antibodies, as part of platform kits and staining conditions, were optimized for each antibody. The clones, dilutions, incubation times, platform and pretreatment for the different stainings were as follows: CD3 – clone LN10, mouse/monoclonal (Novocastra, Leica Biosystems) 1:500, 32 min incubation, Bond Refine DAB kit (Leica Biosystems), pretreatment with ER2 buffer (Tris-EDTA borate, pH 9) for 20 min. CD11b – clone EPR1344, mouse/monoclonal (Abcam) 1:1000, 44 min incubation, ChromoMap Kit (Ventana Medical Systems, Oro Valley, AZ), pretreatment with mild citrate buffer, pH 6 (Ventana Medical Systems), for 30 min. F4/80 – clone BM8, rat/monoclonal (BMA Biomedicals AG, Augst, Switzerland) 1:50, 60 min incubation, ChromoMap Kit (Ventana Medical Systems), pretreatment with protease (#760-2018, 0.38 mg/ml, Ventana Medical Systems) for 4 min. B220 – clone RA3-6B2, rat/monoclonal (Pharmingen, Becton Dickinson, Franklin Lakes NJ) 1:4000, 44 min incubation, ChromoMap Kit (Ventana Medical Systems), pretreatment with Tris-EDTA buffer, pH 9 (Ventana Medical Systems), for 30 min. Collagen IV – rabbit/polyclonal (Cedarlane, Burlington, Canada) incubation 44 min, UltraMap-Rabbit DAB (Ventana Medical Systems), pretreatment with protease (#760-2018, 0.38 mg/ml, Ventana Medical Systems) for 12 min. α-SMA – clone BS66 (Biosite, Solihull, UK) 1:350, incubation 44 min, Bond Refine DAB Rabbit-anti-mouse kit (Leica Biosystems), pretreatment with Tris-EDTA borate, pH 9, for 20 min. Sections from normal murine spleen served as positive control for all immune cell staining.

### RNA-Scope

RNA-Scope analysis was performed on FFPE samples using a 20-base pair *Pkdcc* probe (#516961, Mm-Pkdcc Probe, RNAscope^®^, Advanced Cell Diagnostics, Newark, CA) binding to region 5961655 of *Mus musculus Pkdcc* (NM_134117.2). Positive [peptidylprolyl isomerase B (*PpiB*), pH 6, NM_011149] and negative [dihydrodipicolinate reductase (*DapB*), pH 6, EF191515] controls were run alongside. Sections were processed according to the RNAscope 2.5 HD Reagent Kit protocol (pH 6, PN 322350, Advanced Cell Diagnostics), with the following adjustments: 30 min target retrieval time and 30 min, 40°C, protease plus incubation. Slides were heated using a HybEZ™ Humidity Control Tray in a HybEZ™ oven (Advanced Cell Diagnostics). Sections were counterstained with Hoechst 33342 and mounted with Mowiol^®^. Quantification of red mRNA dots was performed using the analyze particles plug-in of Fiji (Schindelin et al., 2012). Number of of stained mRNA (red) was counted per field of vision.

### Image acquisition and quantification

Histochemical and fluorescence staining were visualized using an Axio-Imager A1 microscope or an Axioskop 2 fluorescence microscope (both from Carl Zeiss, Inc, Oberkochen, Germany) and the corresponding Axiovision software (Carl Zeiss, Inc). Image acquisition was performed either with an Axiocam HRc camera (Carl Zeiss, Inc) connected to the microscope or with a Panoramic 250 slide scanner (3DHISTECH, Budapest, Hungary). Quantifications were performed using either the Fiji (Schindelin et al., 2012) or the Cellprofiler (Carpenter et al., 2006) software.

### Bile isolation from the gallbladder

The gallbladder was removed using forceps and scissors during necropsy. Bile was collected from the gallbladder by simultaneous punctuation with a 20 μl pipette tip and pulling.

### Repeated injections of CCl_4_

Mice were injected intraperitoneally with carbon tetrachloride (CCl_4_) (#319961, Sigma-Aldrich) diluted 1:20 in vehicle [olive oil (v/v in olive oil, 10 µl/g body mass)] or vehicle alone, every third day for 8 weeks as previously described (Weber et al., 2003). Mice were sacrificed 96 h after the last injection.

### Blood plasma analysis

Blood plasma was analyzed using a FUJI DRI-CHEM4000i reader (Fujifilm, Tokyo, Japan), according to the manufacturer's instructions. Briefly, following mouse euthanasia, venous blood was collected into Microvette^®^ Capillary (Li.Hep) collection tubes (#MCVT100-liHEP, Kent Scientific, Torrington, CT) by inserting a cannula into the inferior vena cava. To harvest the plasma, samples were centrifuged for 10 min (2000 ***g***, room temperature). Supernatants were diluted 1:5 in PBS and analyzed on Fuji Dri-Chem alkaline phosphatase ALP-PIII (#8535077, Fujifilm), Fuji Dri-Chem aspartate serum transferase GOT/AST-P III (#8535249, Fujifilm), and Fuji Dri-Chem alanine amino transferase GPT/ALT-P III (#8535261, Fujifilm) slides.

### Isolation of primary hepatocytes and non-parenchymal cells (NPCs)

Isolation of primary hepatocytes and NPCs was performed as previously described (Mohar et al., 2015) with minor modifications. Briefly, following euthanasia of 6-week-old mice, a cannula connected to a perfusion pump was inserted into the inferior vena cava and the portal vein was cut to allow blood to flow out. The liver was washed with Hanks' Balanced Salt Solution (HBSS; #14175053, Thermo Fisher Scientific) supplemented with 25 mM HEPES (#15630106, Thermo Fisher Scientific) and 0.5 mM EGTA and then perfused for 5 min with prewarmed Dulbecco's Modified Eagle's Medium (DMEM) (#31885023, Thermo Fisher Scientific) supplemented with 1% penicillin-streptomycin (P/S; #P4333, Sigma-Aldrich), 15 mM HEPES and 5 mg/ml Liberase™ TM Research Grade (LIBTM-RO, Roche). Subsequently, the gallbladder was removed, and the liver placed in a Petri dish. Liver cells were detached from the surrounding connective tissue by gently shaking and pulling with tweezers. Cells were passed through a 100 µm cell strainer, collected into fresh DMEM supplemented with 10% fetal bovine serum (FBS) (Thermo Fisher Scientific) and P/S and stored on ice for no longer than 15 min. Separation of hepatocytes from NPCs was achieved by centrifugation (2 min, 50 ***g***). The supernatant containing the hepatocyte-depleted NPCs was transferred to a new tube and stored at 4°C. The pelleted hepatocytes were carefully resuspended in isotonic Percoll solution (1:9 ratio of 10× PBS and 100% Percoll^®^, GE Healthcare, Chicago, IL), centrifuged at low speed (2 min, 50 ***g***) and washed with DMEM with 10% FBS and 1% P/S. The final pellet was resuspended in DMEM with 10% FBS and 1% P/S and the cells were plated. NPCs were centrifuged at 750 ***g*** for 10 min and the pellet was gently resuspended in 1 ml PFB (2% FBS, 1 mM EDTA, 1× PBS). The suspension was transferred to a 15 ml tube and the volume adjusted to 2.5 ml of PFB. Next, 2.5 ml of 40% OptiPrep™ (#D1556, Sigma-Aldrich) was added, mixed gently, and the mixture was carefully overlaid with 2 ml PFB. NPCs were harvested from the interphase after centrifugation (1500× ***g***, 25 min) and washed twice in DMEM with 10% FBS and 1% P/S.

### Cell culture

All cells were cultured at 37°C and 5% CO_2_. For trans-differentiation experiments, primary hepatocytes were seeded at a density of 500,000 cells per well of a six-well culture plate or high adhesion Corning^®^ Primaria™ culture plate (#353846, Corning, Corning, NY) and cultured in DMEM with 10% FBS and 1% P/S for 4 h. Following hepatocyte adhesion, cells were washed once with PBS, and the medium changed to Opti-MEM™ I Reduced Serum Medium (#11058021, Thermo Fisher Scientific) supplemented with 1% P/S, with or without 10 μM Y-27632 dihydrochloride (#Y0503, Sigma-Aldrich), 0.5 μM A-83-01 (#SML0788, Sigma-Aldrich) and 3 μM CHIR99021 (#SML1046, Sigma-Aldrich) (collectively referred to as Y/A/C) (Katsuda et al., 2017). Fresh growth medium was added every 48 h for 2–4 weeks. HEK 293T/17 cells (ATCC^®^ CRL-11268™, Manassas, VA) were cultured in DMEM with 10% FBS and 1% P/S. The DNA profile of the cell line was analyzed by Microsynth, Balgach, Switzerland, and confirmed using the PowerPlex^®^ 16 HS System (Promega). Immortalized MEFs were grown in DMEM with 10% FBS and 1% P/S. Cells were regularly tested for mycoplasma contamination using the PCR Mycoplasma Test Kit I/C (#PK-CA91-1048, PromoCell, Heidelberg, Germany). For co-culture experiments, primary hepatocytes were grown in Y/A/C-supplemented medium for 1 week and then plated with MEFs in a 1:10 ratio. The conditioned medium was centrifuged (12,000 ***g***, 10 min) and added to the co-cultures. Validation of VLK expression in the medium was assessed by immunoblotting. Fresh conditioned medium was added every 48 h.

### Cell proliferation assay

Cells were seeded in 96-well plates (5×10^4^ cells/well) and 10 μl of 3-(4,5-dimethylthiazol-2-yl)-2,5-diphenyltetrazolium-bromide (MTT; Sigma-Aldrich, #M-2128) stock solution (5 mg/ml in PBS) in 100 µl DMEM was added to each well. Following a 1 h incubation at 37°C and 5% CO_2_, the supernatant was removed, cells were lysed, and the formazan product was dissolved in 100 μl DMSO. Colorimetric analysis was performed at 560 nm using a GloMax^®^Discover plate reader (Promega).

### 3D cultures in Matrigel

Matrigel^®^ Corning^®^ growth factor-reduced basement membrane matrix (#FAL356230, Chemie Brunschwig AG, Basel, Switzerland) was thawed on ice and mixed 1:1 with Y/A/C-supplemented medium. 20 μl of the suspension was transferred into wells of a 24-well TPP^®^ tissue culture plate (92024, Merck) and allowed to polymerize for 30 min at 37°C. Cells were trypsinized, washed with PBS, seeded in Matrigel^®^ and allowed to expand for 14 days. To stain organoids, Matrigel^®^ was removed from cells using Gentle Cell Dissociation Reagent (0717, Stemcell Technologies, Vancouver, Canada). Images were taken using a ZOE™ Fluorescent Cell Imager (ZOE™ Fluorescent Cell Imager 1450031, BioRad, Hercules, CA).

### Fluorescein diacetate and propidium iodide cell viability determination

Cells were grown in 2D or 3D cultures and stained as described before (Boulos et al., 1999; Lundgren, 1981). After removal of the medium, the staining solution composed of fluorescein diacetate (FDA; C-7521, Sigma-Aldrich, 5 mg/ml), propidium iodide (PI; P4170, Sigma-Aldrich, 2 mg/ml) and 5 ml Opti-MEM™ I Reduced Serum Medium (#11058021, Thermo Fisher Scientific) was freshly prepared and added to the cells. After 15 min incubation at 37°C in the dark, the staining solution was removed, cells were washed with PBS, and new PBS was added to the cells. Images were taken using a ZOE™ Fluorescent Cell Imager (ZOE™ Fluorescent Cell Imager 1450031, BioRad).

### Preparation of paraffin cell blocks

Pelleted cells (750 g, 10 min) were carefully resuspended in four drops of plasma (blood bank of the University Hospital Zurich). One drop of thrombin reagent (60 IU/ml; 100-125, Diagnotec, Liestal, Switzerland) was added to the suspension. After quick agitation, the plasma was allowed to coagulate for 1–2 min. The clot was placed into a cassette containing 4% formalin for at least 10 min and processed using the routine histology program.

### Cell transfection

HEK 293T cells were seeded in six-well plates at approximately 50% confluency and cultured in DMEM with 10% FBS and 1% P/S for 24 h. Transfection with 200 ng pCSX(C)-mPkdcc plasmid (kind gift from C. Yeo, Ewha University, Seoul, South Korea) was performed using the Effectene^®^ Transfection Reagent (#301425, Qiagen, Hilden, Germany). 24 h after transfection, the growth medium was changed to Opti-MEM™ I Reduced Serum Medium supplemented with 1% P/S.

### RNA isolation and qPCR analysis

Total RNA from whole liver tissue or cultured cells was isolated using Trizol^®^ (#15596026, Life Technologies, Carlsbad, CA), according to the manufacturer's instructions, or using the RNA Mini Kit (#IB47303, IBI Scientific, Dubuque, IO). RNA was reverse-transcribed using the iScript™ cDNA synthesis kit (#1708890, BioRad) according to the manufacturer's protocol. Quantitative real-time PCR (qPCR) was performed using the LightCycler^®^480 SYBR Green I Master reaction mix (#04707516001, Roche) on a LightCycler^®^480 II (Roche), and data were evaluated using the LightCycler^®^ 480 software. Details of the qPCR primers are listed in Table S2. Melting curve analysis was performed to exclude non-specific reactions or contaminations. All real-time samples were run in duplicates. Gene expression was determined by the 2^−ΔΔCt^ method and normalized to the expression levels of the gene encoding ribosomal protein S18 (*Rps18*).

### Preparation of protein lysates and western blot analysis

Cells were lysed in lysis buffer (1% NP-40, 150 mM NaCl, 50 mM Tris pH 7.8). Proteins from conditioned media were precipitated with trichloroacetic acid (TCA; #T6399, Sigma-Aldrich; 10% final concentration) and the resulting protein pellet re-suspended in 4% sodium dodecyl sulfate (SDS). Protein concentrations were measured at 560 nm on a GloMax^®^ plate reader using the Pierce Bicinchoninic Acid (BCA) Protein Assay (#23225, Thermo Fisher Scientific) protocol. Protein samples were run on 10% or 12% SDS-PAGE gels and transferred onto a nitrocellulose membrane. The following primary antibodies were used for protein detection: rabbit anti-VLK 289 (1:2000, kindly provided by Malcolm Whitman laboratory, Boston, MA), rabbit anti-TIMP2 (1:2000, #5738, Cell Signaling, Danvers, MA), mouse anti-β-actin (1:5000, #A5441, Sigma-Aldrich), rabbit anti-SOX9 (1:2000, #AB5535, Merck Millipore), rabbit anti-AFP (1:1000, 14550-I-AP, Proteintech), rabbit anti-p-Tyr 1000 (1:2000, #8954, Cell Signaling), mouse anti-total-JNK (1:2000, #sc-7345, Santa Cruz, Santa Cruz, CA) and anti-rabbit IgG coupled to horse radish peroxidase (HRP; 1:5000, #W4018, Promega) or anti-mouse IgG coupled to HRP (1:5000, #W4028, Promega) secondary antibodies. The western Bright ECL kit (#K-12045-D20, Advansta, San Jose, CA) was used to detect the enzymatic HRP reaction product, and membranes were imaged on a Fusion SOLO 6S CCD camera system (Witec AG, Sursee, Switzerland).

### Protein quantification

After SDS-PAGE, polyacrylamide gels were fixed in 40% methanol, 10% acetic acid, 50% ddH_2_O for 30 min, incubated in Coomassie staining solution (0.5% Coomassie Brilliant Blue in ddH_2_O) for 2 h and de-stained (5% methanol, 7% acetic acid, 88% ddH_2_O) overnight at 4°C. Alternatively, to visualize proteins on western blot membranes, Ponceau S solution was used (P7170, Merck).

### Mass spectrometry sample preparation

For mass spectrometry (MS) detection of VLK, primary hepatocytes were transdifferentiated as described above. Protein pellets resulting from TCA-mediated precipitation of proteins from conditioned media or whole cell lysate (WCL) were resuspended in 8 M urea. Proteins were reduced by 1 mM dithiothreitol, alkylated by 5 mM iodoacetamide, and digested by Lys-C (FUJIFILM Wako Pure Chemical Corporation, 129-02541) for 4 h. The concentration of urea was diluted to 1 M before overnight trypsin (Promega, V5113) digestion. The peptides were acidified by trifluoroacetic acid (TFA) and purified by HR-X Column (Macherey-Nagel, 730936P45). After elution with 0.1% formic acid in 80% acetonitrile, the peptides were dried in lyophilizer overnight. Peptides were resuspended in 20 μl of 0.1% formic acid. Peptide concentration was determined by BCA assay using Pierce BCA Protein Assay Kit (Thermo Scientific, 23225).

Liquid chromatography tandem mass spectrometry (LC-MS/MS) measurements were performed on a QExactive (QE) Plus and HF-X mass spectrometer coupled to an EasyLC 1000 and EasyLC 1200 nanoflow-HPLC, respectively (all Thermo Scientific). Peptides were fractionated on a fused silica HPLC-column tip [I.D. 75 μm, New Objective, self-packed with ReproSil-Pur 120 C18-AQ, 1.9 μm (Dr. Maisch) to a length of 20 cm] using a gradient of A (0.1% formic acid in water) and B (0.1% formic acid in 80% acetonitrile in water). Samples were loaded with 0% B with a maximum pressure of 800 Bar, and peptides were separated by 5%–30% B within 180 min with a flow rate of 250 nl/min. Spray voltage was set to 2.3 kV and the ion-transfer tube temperature to 250°C; no sheath and auxiliary gas were used. Mass spectrometers were operated in the data-dependent mode; after each MS scan (mass range m/z=370–1750; resolution: 70,000 for QE Plus and 120,000 for HF-X), a maximum of ten or twelve MS/MS scans were performed using a normalized collision energy of 25%, a target value of 1000 (QE Plus)/5000 (HF-X) and a resolution of 17,500 for QE Plus and 30,000 for HF-X. MS raw files were analyzed using MaxQuant (version 1.6.2.10) (Cox and Mann, 2008) using a Uniprot full-length *Mus musculus* database (April, 2016) and common contaminants such as keratins and enzymes used for in-gel digestion as reference. Carbamidomethylcysteine was set as fixed modification and protein amino-terminal acetylation, and oxidation of methionine were set as variable modifications. Label-free quantification (LFQ) was selected for normalized protein quantification (Cox et al., 2014). The MS/MS tolerance was set to 20 ppm and three missed cleavages were allowed using trypsin/P as enzyme specificity. Peptide, site and protein false discovery rate (FDR) based on a forward-reverse database were set to 0.01, minimum peptide length was set to 7, the minimum score for modified peptides was 40, and minimum number of peptides for identification of proteins was set to one, which must be unique. The ‘match-between-run’ option was used with a time window of 0.7 min.

### METAVIR scoring

The METAVIR was used to stage the degree of fibrosis. This scoring defines activity and fibrosis for chronic viral hepatitis in humans (A=0,1,2,3: none, mild, moderate, severe) and fibrosis (F=0,1,2,3,4: no fibrosis, portal fibrosis, bridging fibrosis with few septae, bridging fibrosis with many septae, and cirrhosis) (Bedossa and Poynard, 1996).

### Bile acid measurement

Bile acids were quantified by LC-MS/MS according to the principle described previously (Scherer et al., 2009; Krautbauer et al., 2016). A Kinetex^®^ 2.6 µm Biphenyl 50×2.1 mm column was used to cover also muricholic acids and their conjugates. For most of the analytes, stable isotope-labeled internal standards were added prior to acetonitrile precipitation. Liver homogenates corresponding to 2 mg wet mass were subjected to analysis. Bile was analyzed after 1000-fold dilution. In brief, for all analytes, stable isotope-labeled internal standards were added prior to acetonitrile precipitation.

### Statistical analyses

Statistical analyses were performed using Prism 8 software (Graphpad Software Inc, San Diego, CA). Quantitative data are expressed as mean±s.d. Differences between groups (i.e. genotypes) were determined using one-way ANOVA or two-way ANOVA in the case of treatment studies. In some cases, Tukey's multiple comparison test was applied as detailed in the figure legends. Comparisons among two groups were made using unpaired, non-parametric Mann–Whitney test. *P≤0.05, **P≤0.01, ***P≤0.001, ****P≤0.0001.

## Supplementary Material

Supplementary information

Reviewer comments
