# Supplementary material for: Vertebrate lonesome kinase modulates the hepatocyte secretome to prevent perivascular liver fibrosis and inflammation
Source: J Cell Sci. 2022 Apr 12;135(7):jcs259243. doi: 10.1242/jcs.259243 (PMC9016620; doi:10.1242/jcs.259243)
Supplement: Supplementary information [file joces-135-259243-s1.pdf]

Supplementary Figure 1

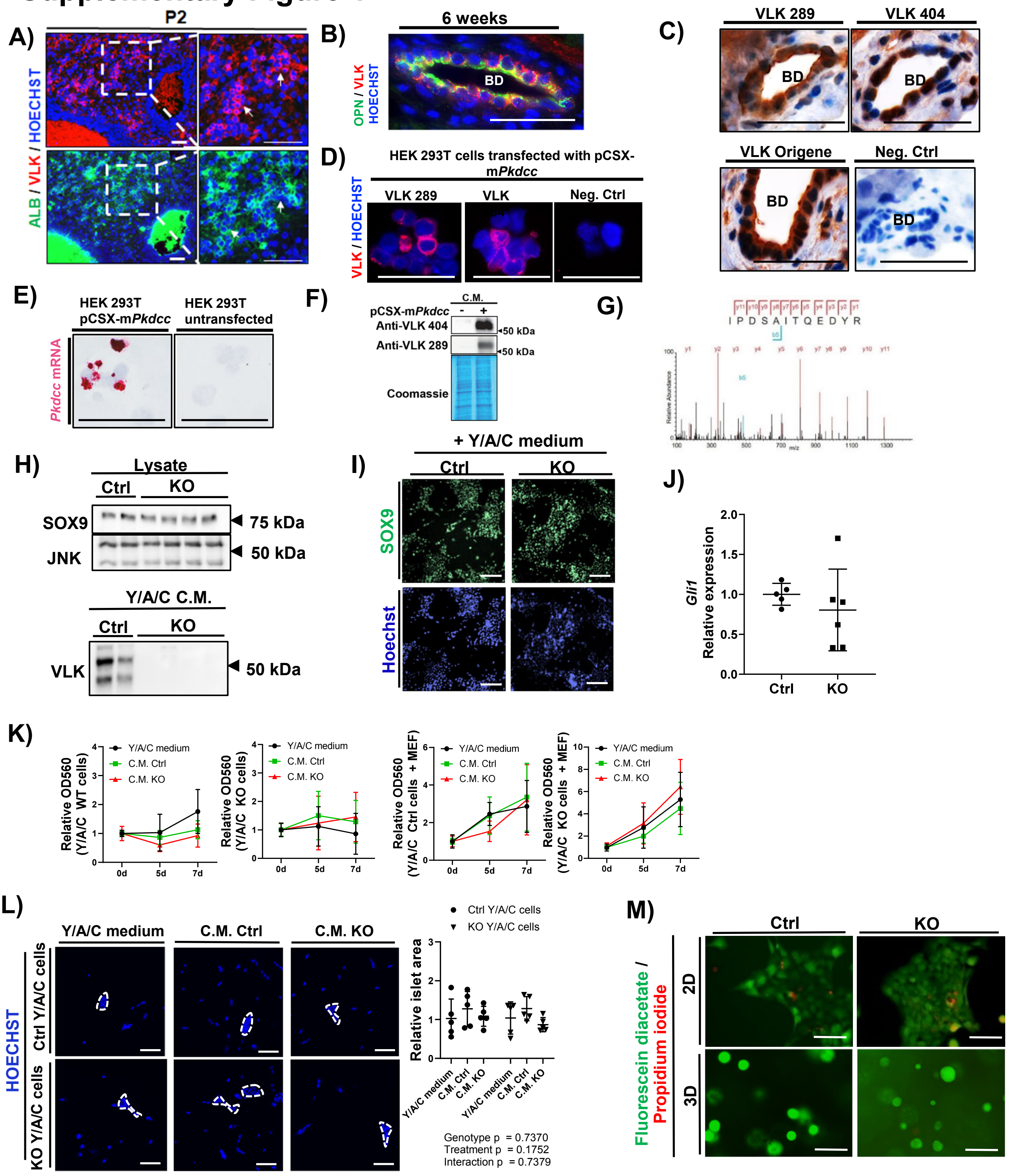

Journal of Cell Science • Supplementary information

Fig. S1. Validation of the specificity of VLK immunostaining and characterization of primary liver progenitor-like cells that secrete VLK

(A) Serial mouse liver sections at P2 were analyzed by immunofluorescence for VLK (red) and albumin (Alb; green). Nuclei were counterstained with Hoechst (blue). The area indicated by the white rectangle in the left panel is shown at higher magnfication in the right panel. Arrows point to ALB/VLK-positive hepatocytes.

(B) Mouse liver sections were analyzed by immunofluorescence for VLK (red) and osteopontin (OPN; green). Nuclei were counterstained with Hoechst (blue). BD: Bile duct.

(C) Liver sections were analyzed by immunohistochemistry for VLK using three different antibodies (VLK 289, VLK 404, VLK Origene). Sections were counterstained with hematoxylin. BD = Bilde duct.

(D) HEK 293T cells were transfected with a VLK expression vector (pCSX-*mPkdcc*) and analyzed by immunofluorescence using two different VLK antibodies (red) (VLK 289 and VLK 404) or no first antibody (negative control). Nuclei were counterstained with Hoechst (blue).

(E) HEK 293T cells, transfected or not with a VLK expression vector (pCSX-*mPkdcc*), were analyzed by RNAscope for *Pkdcc* mRNA (red).

(F) HEK 293T cells were transfected with a VLK expression plasmid (pCSX-m*Pkdcc*) or left untransfected. Conditioned media (C.M.) produced in the presence of serum-free Opti-MEM were analyzed for VLK by Western blot using two different VLK antibodies. Coomassie staining was used to verify equal loading.

Journal of Cell Science • Supplementary information

(G) Representative spectrum of a unique VLK peptide identified by mass spectrometry analysis of the secretome of cells grown in Y/A/C-supplemented medium. Spectrum is representative for 3 independent batches of C.M. VLK peptide was not detected in C.M. of KO cells.

(H) rimary hepatocytes from Ctrl or KO mice were cultured in Y/A/C-supplemented medium for 14 days. C.M. were analyzed by Western blot for VLK, and lysates were analyzed for or SOX9 and JNK. Representative of 3 independent experiments.

(I) SOX9 expression (green) detected by immunofluorescence staining of primary hepatocytes from Ctrl or KO mice grown in Y/A/C-supplemented medium. Nuclei were stained with Hoechst (blue). Representative images of 3 independent isolations are shown.

(J) RNA samples from Y/A/C Ctrl or KO cells cultured in Y/A/C-supplemented medium for 14 days were analyzed by RT-qPCR for *Gli1* relative to *Rps18*. Ctrl: N=5, KO: N=6. Mean expression levels in Ctrl cells were set to 1.

(K) Primary hepatocytes from Ctrl and KO mice were cultured for 7 days in the indicated medium in the absence (left two panels) or presence of immortalized mouse embryonic fibroblasts (MEF; right two panels). N=6-12 cultures from three independent experiments. Values obtained at day 0 were set to 1.

(L) Representative photomicrographs of primary hepatocytes from Ctrl or KO mice incubated in the presence of the indicated culture medium and analyzed by staining of nuclei with Hoechst. Graph shows average area of “liver progenitor” islets. Results of three independent cell culture experiments are shown (right panel), each dot represents

Journal of Cell Science • Supplementary information

a culture from a different mouse. N=5 per group. Mean islet area of Ctrl cells cultured in Y/A/C medium was set to 1.

(M) Representative immunofluorescence images of primary hepatocytes from Ctrl or KO mice grown in 2D or 3D incubated with Y/A/C medium for 10 days. Cell viability was controlled by addition of fluorescein diacetate (green) and propidium iodide (red). N=3.

Graphs show mean ± SD. Statistical significance was assessed by unpaired nonparametric Mann-Whitney test (J), ordinary one-way ANOVA (Tukey's multiple comparisons test) (K) or two-way ANOVA (L). Magnification bars: 100 μm.

Journal of Cell Science • Supplementary information

Supplementary Figure 2

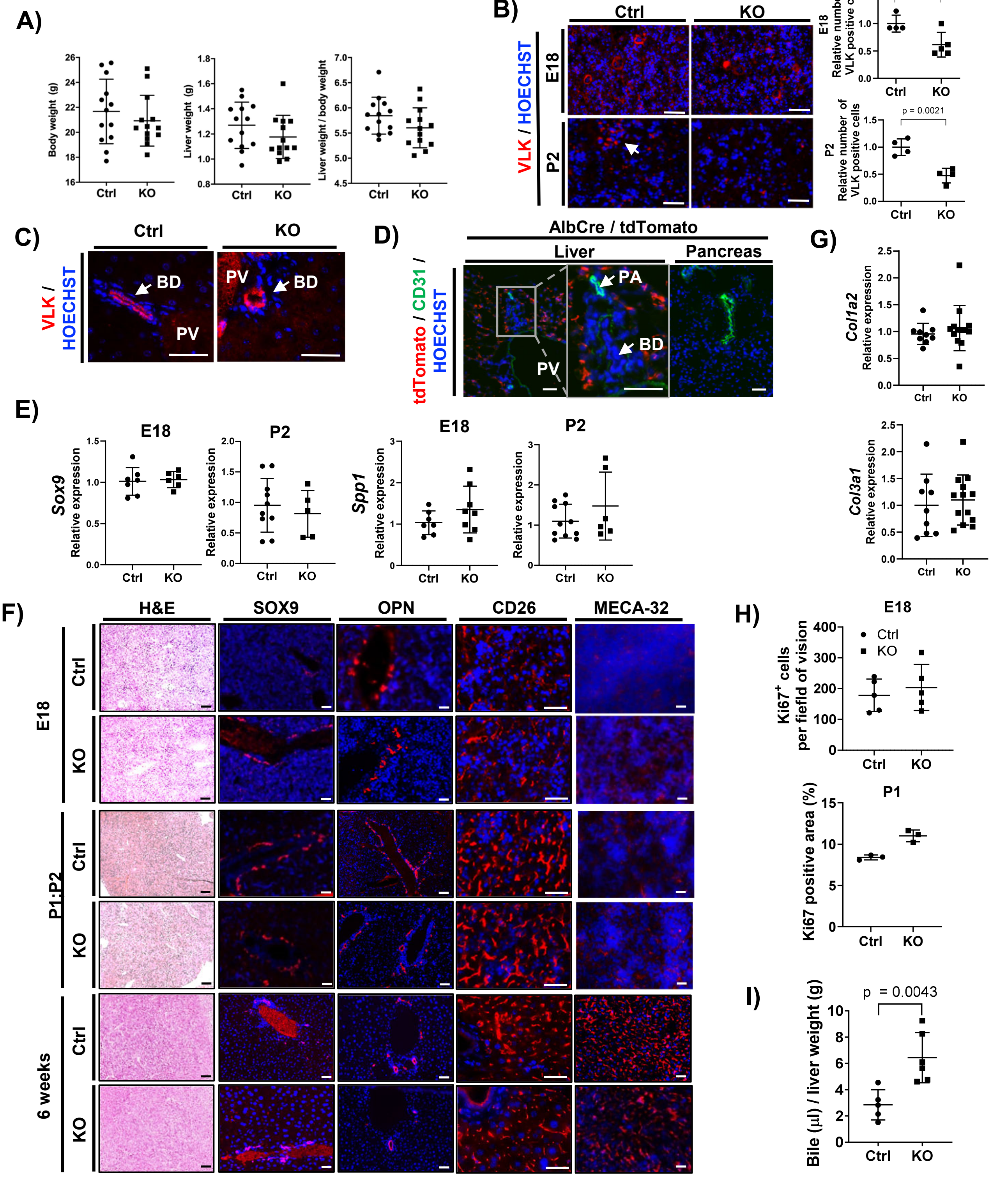

Journal of Cell Science • Supplementary information

Fig. S2. Characterization of liver morphology and function in mice lacking VLK in hepatocytes

(A) Body weight, liver weight and liver-to-body weight ratio in 6 week-old Ctrl and KO mice.

(B) Immunofluorescence detection of VLK (red) in liver sections of Ctrl and KO mice and counterstaining of nuclei with Hoechst (blue). Graph shows quantification of VLK-positive cells at the indicated developmental stage. N = 4-5 per genotype.

(C) Immunofluorescence staining of liver sections from adult mice for VLK (red) and counterstaining of nuclei with Hoechst (blue). BD: bile duct, PV: portal vein.

(D) Fluorescence detection of tdTomato combined with immunofluorescence staining for CD31 in liver and pancreas sections of double-transgenic mice expressing Cre under the control of the albumin promoter and harboring a tdTomato reporter gene preceded by a floxed STOP cassette. Note the specific deletion of the cassette in hepatocytes, but not in cholangiocytes. PA: portal artery.

(E) RNA from liver tissue of Ctrl or KO mice at the indicated age was analyzed by RT-qPCR for *Sox9 (upper panels)* and *Spp1* (lower panels) relative to *Rps18*. E18: N=6-7, P2: N=5-10. Mean expression levels in Ctrl mice were set to 1.

(F) Liver sections from Ctrl or KO mice at the indicated age were stained with H&E or analyzed by immunofluorescence for SOX9, OPN, CD26 and MECA-32 (red). Nuclei were counterstained with Hoechst (blue).

(G) RNA from liver tissue of adult Ctrl or KO mice was analyzed by RT-qPCR for *Col1a2* and *Col3a1* relative to *Rps18*. N = 9-12. Mean expression levels in Ctrl mice were set to 1.

(H) Quantification of Ki67-positive cells in the liver of E18 and P1 old mice. E18: N=5, P1: N=3.

(I) Volume of bile per gram liver weight in the gallbladders of Ctrl or KO mice. N = 5-6. Graphs show mean ±SD. Statistical significance was assessed in all panels of this figure by unpaired nonparametric Mann-Whitney test. Magnification bars: 100 μm.

Supplementary Figure 3

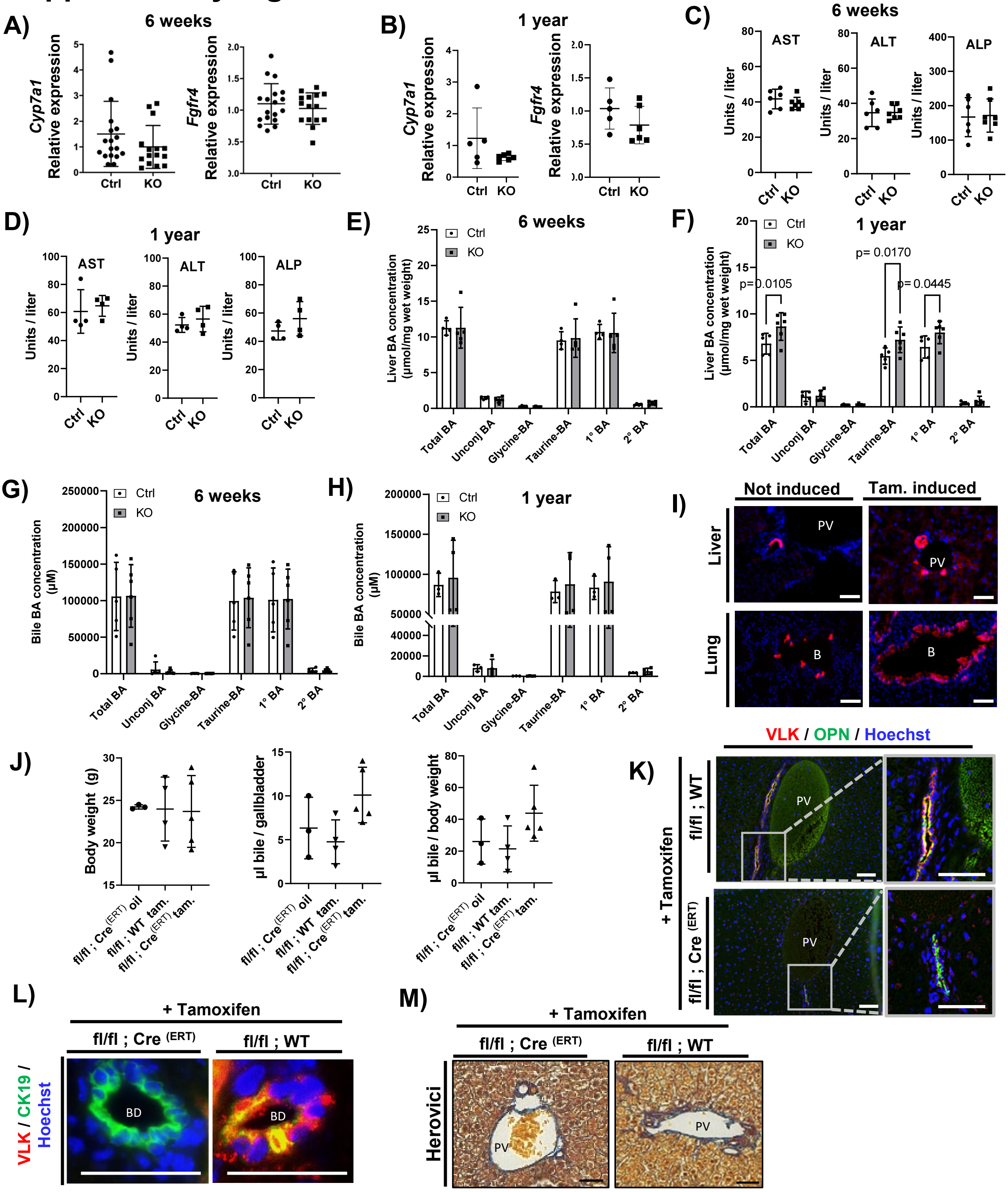

Journal of Cell Science • Supplementary information

Fig. S3. Hepatocytes but not cholangiocyte-derived VLK is important for liver homeostasis

(A, B) RNA from liver tissue of 6-week (A) or 1-year old (B) Ctrl or KO mice was analyzed by RT-qPCR for *Cyp7a1* and *Fgfr4* relative to *Rps18*. N=15-18. Mean expression levels in Ctrl mice were set to 1.

(C, D) AST, ALT and ALP enzyme levels in the plasma of 6-week (C) or 1 year-old (D) Ctrl and KO mice. N=6-7.

(E, F) Liver total bile-acid (BA), unconjugated (unconj) BA, glycine-conjugated BA, taurine-conjugated BA, primary (1°) BA and secondary (2°) BA concentrations were determined in 6 week-old liver (E) or 1 year-old liver (F). White bars represent Ctrl, grey bars KO mice. Graphs show mean ±SD, N=3-6. Statistical analysis was performed using 2-way ANOVA, Sidak's multiple comparisons test.

(G, H) Bile from the gallbladder of 6 week-old mice (G) and 1 year-old mice (H) was analyzed by liquid chromatography-tandem mass spectrometry. White bars represent Ctrl, grey bars KO mice. Data show mean ±SD, N=3-6. Statistical analysis was performed using 2-way ANOVA, Sidak's multiple comparisons test.

(I) Representative photomicrographs of sections from mouse liver carrying a tdTomato Cre reporter and expressing a CK19Cre$^{(ERT)}$ transgene. Nuclei were counterstained with Hoechst (blue). Mice were injected with tamoxifen to activate the Cre$^{(ERT)}$ promoter (Tam. induced) or oil (vehicle) (not induced). PV = portal vein, B = bronchiole.

(J) Body weight and bile volume in fl/fl;Cre$^{(ERT)}$ or fl/fl;Wt mice injected with tamoxifen or oil. N=3-5.

(K) Representative photomicrographs of sections from fl/fl;Cre$^{(ERT)}$ or fl/fl;Wt mice injected with tamoxifen and stained for VLK (red) and osteopontin (OPN) (green). Nuclei were counterstained with Hoechst (blue). N=4. PV = portal vein.

(L) Representative photomicrographs of liver bile ducts from fl/fl;Cre$^{(ER)}$ or fl/fl;Wt mice injected with tamoxifen and stained for VLK (red) and cytokeratin 19 (CK19) (green). Nuclei were counterstained with Hoechst (blue). N=4. BD = bile duct.

(M) Representative liver sections from fl/fl;Cre$^{(ERT)}$ or fl/fl;Wt mice injected with tamoxifen, stained with Herovici. N=4. PV = portal vein.

Journal of Cell Science • Supplementary information

Supplementary Figure 4

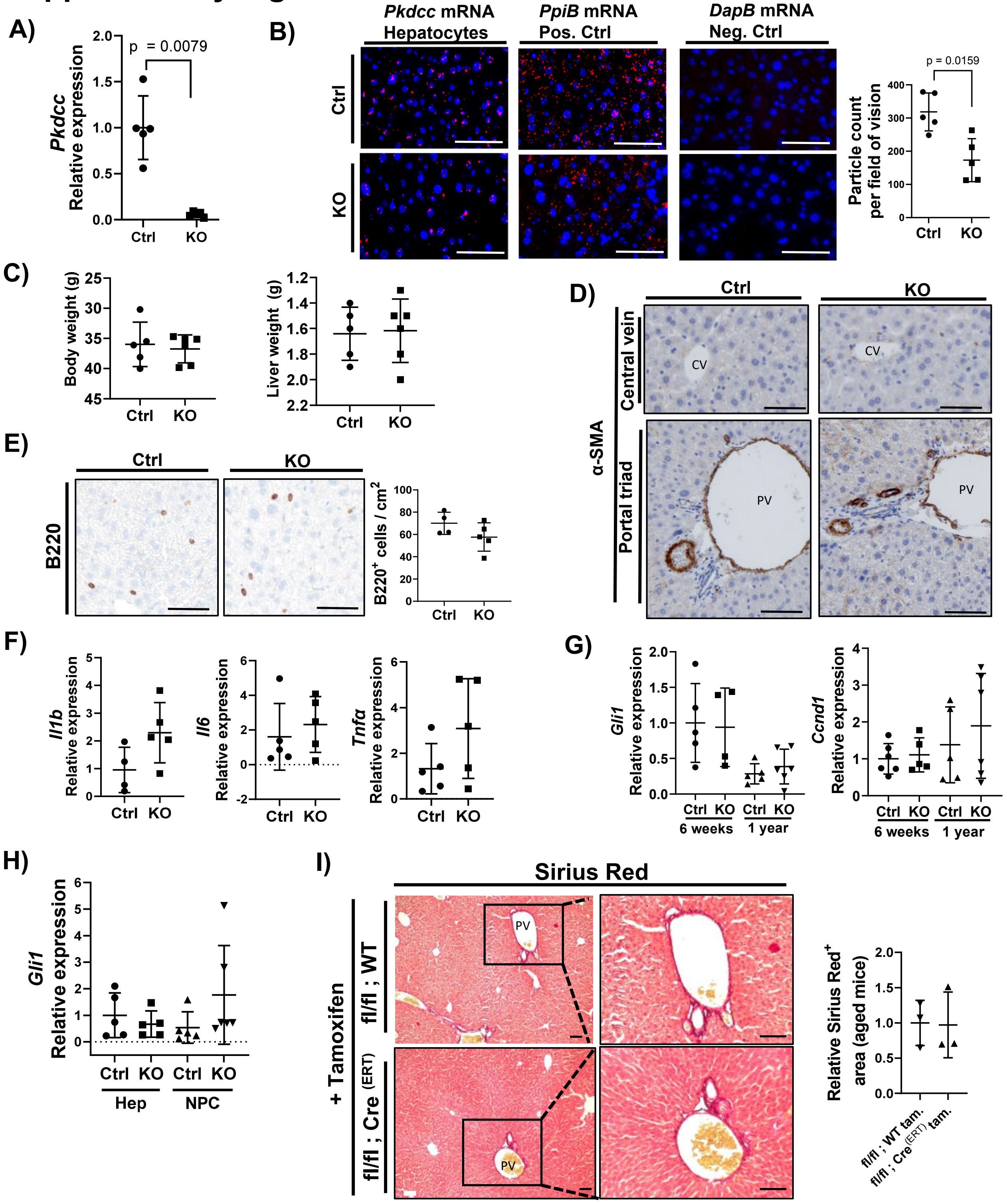

Journal of Cell Science • Supplementary information

Fig. S4. Characterization of liver function in aged mice lacking VLK in hepatocytes.
(A) RNA from total liver of 56 week-old Ctrl and KO mice was analyzed by RT-qPCR for *Pkdcc* relative to *Rps18*. N= 5-6. Mean expression levels in Ctrl mice were set to 1. (B) Pkdcc mRNA in liver of 56 week-old Ctrl and KO mice visualized by RNA-Scope *in situ* hybridization (red). PpiB and DapB mRNAs were used as positive and negative controls, respectively. Nuclei were counterstained with Hoechst. Graph shows quantification of Pkdcc RNA positive particles with N=5.

(C) Body and liver weight of 1 year-old Ctrl and KO mice. N=5-6.

(D) Liver sections were analyzed by immunohistochemistry for α-SMA. Central vein (CV) in the upper panels and portal vein (PV) in the lower panels are depicted. Sections were counterstained with hematoxylin.

(E) Representative photomicrographs of liver sections analyzed by immuno-histochemistry for B220 and quantification of B220-positive cells. N=4-5; n=3 pictures per mouse. Mean in Ctrl mice was set to 1.

(F) RNA samples from whole liver of wild-type mice at 56 weeks of age were analyzed for *Il1b, Il6,* and *Tnfa* relative to *Rps18* expression by RT-qPCR. N=4-5 mice per time point.

(G) RNA from total liver of 6-week-old and 56 week-old Ctrl and KO mice was analyzed by RT-qPCR for *Gli1* and *Cnnd1* relative to *Rps18*. N= 4-6 mice per genotype and age. Mean expression levels in Ctrl mice were set to 1.

(H) RNA from total primary hepatocytes and non-parenchymal cells (NPC) of 6 week-old Ctrl and KO mice was analyzed by RT-qPCR for *Gli1* relative to *Rps18*. N= 5-6 mice per genotype. Mean expression levels in Ctrl mice were set to 1.

(I) Representative liver sections from fl/fl;Cre$^{(ERT)}$ or fl/fl;Wt mice injected with tamoxifen over 8 weeks and analyzed at the age of 42-weeks. Sections were stained with Sirius Red. Graph shows quantification of relative Sirius Red positive area per field of vision. fl/fl;Wt aged: N=3, fl/fl;Cre$^{(ERT)}$ aged: N=3. PV = portal vein.

Graphs represent mean ±SD. Statistical significance was assessed by unpaired non-parametric Mann-Whitney test. Magnification bars: 100 μm.

Journal of Cell Science • Supplementary information

Supplementary Figure 5

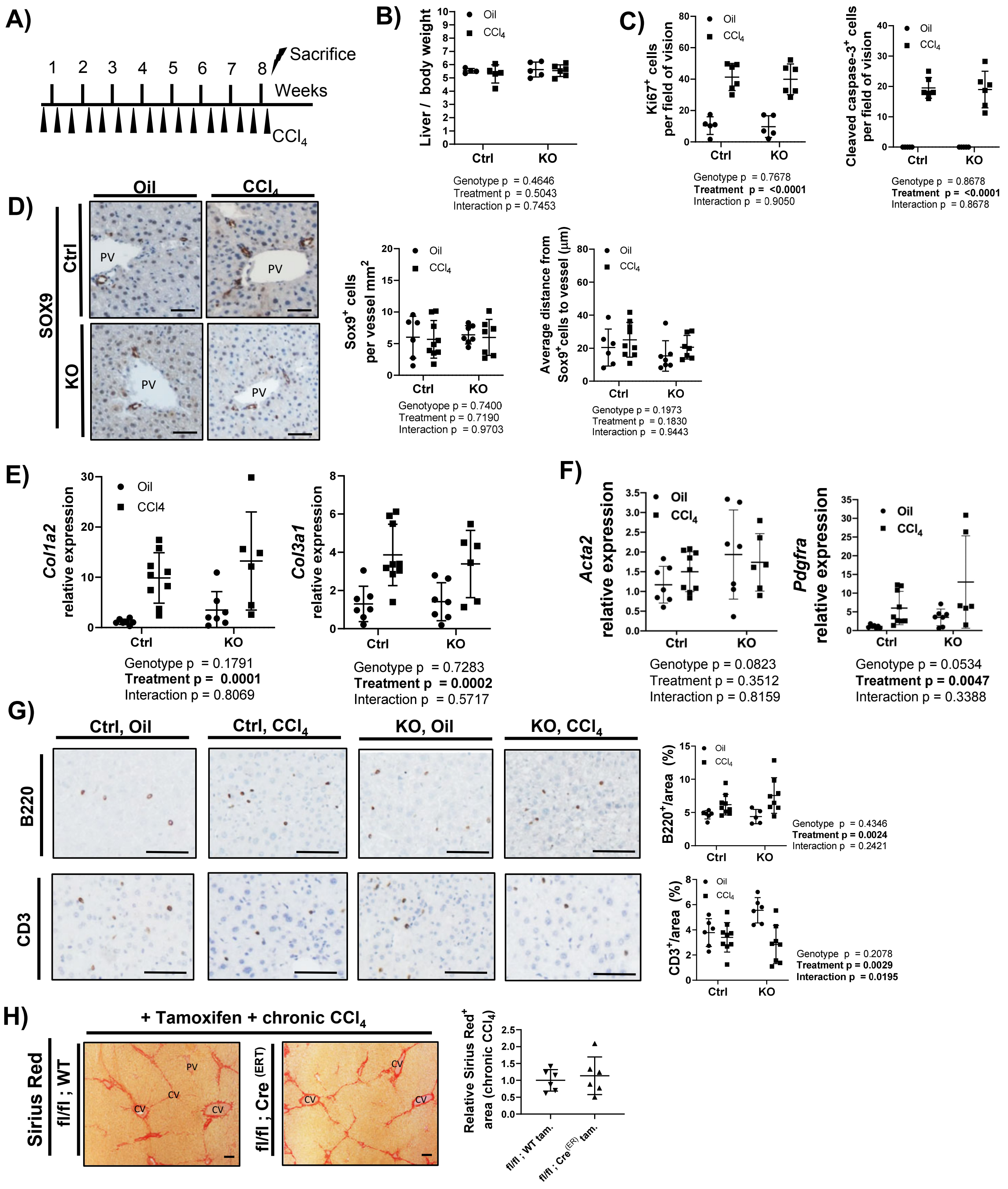

Fig. S5. Analysis of liver fibrosis in Ctrl and KO mice chronically treated with CCl_4

(A) Timeline of CCl_4 injections. Injection with vehicle (olive oil) was used as control.

(B) Liver to body weight ratios in Ctrl and KO mice following chronic oil or CCl_4 treatment. Ctrl oil: N=4; Ctrl CCl_4: N=5; KO oil: N=5; KO CCl_4: N=6.

(C) Graph showing quantification of Ki67- or cleaved caspase 3-positive cells per field of vision in mice chronically treated with CCl_4 or oil, respectively. N=5-6 per group.

(D) Representative photomicrographs of liver sections analyzed by immunohistochemistry for SOX9 and quantification (right panels) of $SOX9^+$ cells per vessel area and of average distance of $SOX9^+$ cells from vessels. N=6-9 mice, = 3 pictures per mouse. PV = portal vein.

(E, F) RNA samples from total liver of Ctrl and KO mice, chronically treated with CCl_4 or vehicle, were analyzed by RT-qPCR for *Col1a2* and *Col3a1* (E) or *Acta2* and *Pdgfra* (F) relative to *Rps18*. Ctrl oil: N=3-7; Ctrl CCl_4: N=5-9; KO oil: N=5-7; KO CCl_4: N=5-6. Mean expression levels in Ctrl mice were set to 1.

(G) Representative liver sections from Ctrl and KO mice chronically treated with CCl_4 or vehicle (oil), analyzed by immunohistochemistry for B220 or CD3, co-stained with hematoxylin, and quantification (right panels) of the $B220^+$ and $CD3^+$ cells per area. Ctrl oil: N=6-7; Ctrl CCl_4: N=5-9; KO oil: N=6; KO CCl_4: N=6.

H) Representative liver sections from fl/fl;$Cre^{(ERT)}$ or fl/fl;Wt mice injected with tamoxifen and chronically treated with CCl_4 or oil (vehicle) over 8 weeks stained with Sirius Red. Graph shows quantification of relative Sirius Red positive area per field of vision. fl/fl;Wt + CCl_4: N=6, fl/fl;$Cre^{(ERT)}$ + CCl_4: N=6. PV = portal vein, CV = central vein.

Graphs show mean ±SD. Statistical analysis was performed using two-way ANOVA (B, C, D, E, F, G) or unpaired non-parametric Mann-Whitney test (H). Representative results from two independent experiments are shown. Magnification bars: 100 µm.

Journal of Cell Science • Supplementary information

Supplementary Figure 6

A)

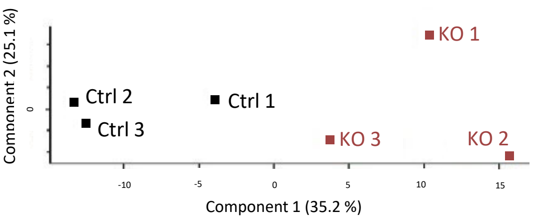

B)

Access. No.	Protein Name	Fold Change	p-value
Q9JHI0	Mmp19	3.33	0.035
P02772	Afp	2.47	0.020
Q05117	Acp5	2.46	0.016
P02469	Lamb1	2.21	0.013
Q9CYN9	Atp6ap2	1.90	0.008
P02468	Lamc1	-4.36	0.026
P10493	Nid1	-3.91	0.006
P98063	Bmp1	-3.16	0.046
Q05793	Hspg2	-2.28	0.020
P11276	Fn1	-2.19	0.028
P09803	Cdh1	-1.94	0.031
Q99M71	Epdr1	-1.64	0.010
P54818	Galc	-1.53	0.015
Q6GV12	Kdsr	-1.52	0.034

C)

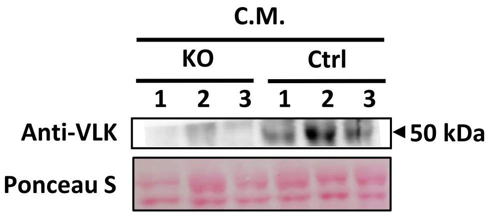

D)

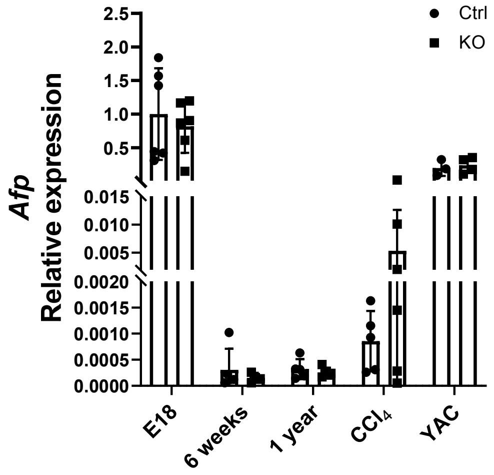

Fig. S6. Increased AFP levels in the conditioned medium (C.M.) and whole cell lysate (WCL) of Y/A/C treated hepatocytes

(A) Principle component analysis (PCA) to identify variability in the dataset. Individuals are represented by black squares (Ctrl) or red squares (KO).

(B) LC-MS/MS list of proteins with a signal peptide that are significantly differentially abundant (FC>1.5, p<0.05) in WCL of Y/A/C-treated hepatocytes. Red: more abundant in the KO cells; green; less abundant in the KO cells. N=3 per genotype.

Journal of Cell Science • Supplementary information

(C) Representative Western blot of conditioned media (C.M.) of Y/A/C-supplemented hepatocytes analyzed for VLK. Ponceau S was used to ensure equal loading. Every line represents a cell isolation from an individual mouse.

(D) NA from total liver of E18, 6-week-old and 1-year-old Ctrl and KO mice and from CCl_4-treated Ctrl and KO mice, or from Y/A/C treated hepatocytes of Ctrl and KO mice were analyzed by RT-qPCR for *Afp* relative to *Rps18*. N= 5-6 mice per genotype. Mean expression levels in Ctrl mice at E18 were set to 1.

Graphs show mean ± SD. Statistical significance was assessed by unpaired non-parametric Mann-Whitney test.

Journal of Cell Science • Supplementary information

Table S1. Genotyping

The following primers were used:

Primer	Sequence forward primer	Sequence reverse primer
mPkdcc	CAC ACG CTC AAT CAT ACC ACA	GGT CAT TAG GTC ACA GGG TAG
mCre	CGA CCA GGT TCG TTC ACT CA	CGA GTT GAT AGC TGG CTG GT
mR26 loc wt	AAG GGA GCT GCA GTG GAG TA	CCG AAA ATC TGT GGG AAG TC
mtdTomato	CTG TTC CTG TAC GGC ATG G	GGC ATT AAA GCA GCG TAT
mCK19	CAG AAT GCG CAG GAA TTG AC	mCK19wt CGG AAA AAA CCC CCT GA
		mCk19mut AGG CAA ATT TTG TGT AGG G

Table S2. Primer list RT-qPCR

Primer	Sequence forward primer	Sequence reverse primer
mPkdcc	CACACGCTCAATCATACCACA	GGTCATTAGGTCACAGGGTAG
mAlb	CGACCAGGTTCGTTCACTCA	CGAGTTGATAGCTGGCTGGT
mSox9	GCTTGTCCGTTCTTCACCGA	TCTGGAGGCTGCTGAACGAG
mAdgre1	GACAAACACTTGGTGGTGTGA	CCAGAATCCAGTCTTTCCCA
mCol1a	TGTTCAGCTTTGACCTCCGGC	TCTCCCTTGGGTCCCTCG
mCol3a1	TCCCCTGGAATCTGTGAATC	TGAGTCGAATTGGGGAGAAT
mPdgfra	GTGCGACCTCCAACCTGA	GGCTCATCTCACCTCACATCT
mActa2	CTGCCGAGCGTGAGATTG	ATAGGTGGTTTCGTGGATGC
mCyp7a1	GGGATTGCTGTGGTAGTGAGC	GGTATGGAATCAACCCGTTGTC
mFgfr4	TTGGCCCTGTTGAGCATCT	GCCCTCTTTGTACCAGTGACG
mSpp1	CTTTCACCGGGAGGGAGGA	TGCAGTTCTCCTGGCTGAAT
mTgfb	AAAGCCCTGTATTCCGTCTCC	CGCAACAACGCCATCTATGA
m18s	GATCCATTGGAGGGCAAGTCT	CCAAGATCCAACTACGAGCTTTT
mGli1	GTATGAGACAGACTGCCGCT	T GCTCACTGTTGATGTGGTGC
mCcnD1	ACTGCCGAGAAGTTGTGCAT	AAGCAGTTCCATTTGCAGCAG
mAfp	TGCTTCCAGACAAAGAGAGCAT	GTTGTTGCCTGGAGGTTTCG
