# Supplementary material for: Vertebrate lonesome kinase modulates the hepatocyte secretome to prevent perivascular liver fibrosis and inflammation
Source: J Cell Sci. 2022 Apr 12;135(7):jcs259243. doi: 10.1242/jcs.259243 (PMC9016620; doi:10.1242/jcs.259243)
Supplement: Reviewer comments [file joces-135-259243_review_history.pdf]

Vertebrate lonesome kinase modulates the hepatocyte secretome to prevent perivascular liver fibrosis and inflammation

Sophia Pantasis, Juliane Friemel, Salome Mirjam Brütsch, Zehan Hu, Sabrina Krautbauer, Gerhard Liebisch, Joern Dengjel, Achim Weber, Sabine Werner and Mattia Renato Bordoli
DOI: 10.1242/jcs.259243

Editor: Kathleen Green

Review timeline

Original submission:	12 August 2021
Editorial decision:	4 October 2021
First revision received:	2 February 2022
Accepted:	8 March 2022

Original submission

First decision letter

MS ID#: JOCES/2021/259243

MS TITLE: Vertebrate Lonesome Kinase modulates the hepatocyte secretome to prevent perivascular liver fibrosis and inflammation

AUTHORS: Sophia Pantasis, Juliane Friemel, Salome Bruetsch, Zehan Hu, Sabrina Krautbauer, Gerhard Liebisch, Joern Dengjel, Achim Weber, Sabine Werner, and Mattia Bordoli
ARTICLE TYPE: Research Article

We have now reached a decision on the above manuscript.

To see the reviewers' reports and a copy of this decision letter, please go to: https://submit-jcs.biologists.org and click on the 'Manuscripts with Decisions' queue in the Author Area. (Corresponding author only has access to reviews.)

As you will see, the reviewers' opinions are somewhat divergent, but collectively they raise a number of substantial criticisms that prevent me from accepting the paper at this stage.

Referee #1 raises the concern, which I share, that the studies may not appropriate for the JCS readership based on the fact that, while the work is quite substantive, it fails to provide sufficient mechanistic insight into the reported observations. The studies are largely descriptive in nature and reveal complex phenotypes without any mechanistic details of VLK function. One example is that the title of the paper states that VLK modulates the hepatocyte secretome. However, as stated by referee #1, the experiments shown in Fig. 2 to support that statement are indirect. Additionally, VLK is highly regulated during development being expressed in high levels in embryonic hepatocytes then shifting to a bile duct distribution in adults. A hepatocyte-specific KO approach was chosen and it is not clear how the phenotypes relate to this specific KO condition. A number of questions arise related to this issue, which referee #1 nicely outlines in their review. While these questions are more than can be addressed in a single manuscript, some additional depth would be necessary to make the work of broad interest to the JCS audience. At the present time, the work is better suited for a journal targeting translational/clinical liver scientists or other GI researchers.

© 2022. Published by The Company of Biologists under the terms of the Creative Commons Attribution License (https://creativecommons.org/licenses/by/4.0/).

While you may wish to submit this work elsewhere so as not to delay publication, I have not rejected the manuscript outright, to give you the opportunity to decide whether you would be interested in developing mechanistic aspects of the study further with the idea of targeting the work to the JCS audience.

Please let us know what you decide. We would send the paper back to the same referees for re-review.

We are aware that you may be experiencing disruption to the normal running of your lab that makes experimental revisions challenging. If it would be helpful, we encourage you to contact us to discuss your revision in greater detail. Please send us a point-by-point response indicating where you are able to address concerns raised (either experimentally or by changes to the text) and where you will not be able to do so within the normal timeframe of a revision. We will then provide further guidance. Please also note that we are happy to extend revision timeframes as necessary.

Is you do decide to submit a revised version, please ensure that you clearly highlight all changes made in the revised manuscript. Please avoid using 'Tracked changes' in Word files as these are lost in PDF conversion.

I should be grateful if you would also provide a point-by-point response detailing how you have dealt with the points raised by the reviewers in the 'Response to Reviewers' box. Please attend to all of the reviewers' comments. If you do not agree with any of their criticisms or suggestions please explain clearly why this is so.

<u>Reviewer 1</u>

Advance summary and potential significance to field

This manuscript provides extensive phenotypic characterization of mice with a liver-specific knockout of vertebrate lonesome kinase (VLK)- the only know secreted tyr kinase. The phenotypes (albeit complex) would likely be interesting to those clinical/translational researchers actively studying liver disease (especially fibrosis) or liver regeneration.

Comments for the author

This manuscript examines the mouse phenotypes of hepatocyte-specific knock out of Vertebrate Lonesome Kinase (VLK). VLK is the only known secreted tyrosine kinase - an inherently interesting property - and from studies in other tissues, VLK has been shown to phosphorylate a host of proteins both in the secretory pathway and in the extracellular milieu. It has not been studied in liver - so the premise for these studies is interesting. The manuscript represents an enormous body of work both in the 5 figures within the manuscript itself and the 6 supplementary figures. The experiments were performed with rigor and the appropriate controls, statistical analysis and are informative. The results are likely quite interesting to those actively studying liver disease and regeneration and to physician scientists.

The concern is that the studies may not appropriate for the JCS readership where mechanistic, cell biological studies are highlighted. The studies described in this manuscript are largely descriptive in nature and reveal phenotypes (many of which are interesting, but very complex) without any mechanistic details of VLK function. For example, the title of the paper states VLK modulates the hepatocyte secretome. However, the experiments shown in Fig. 2 to support that statement are indirect - no alterations in the secretome were specifically examined (e.g., the profile of secreted proteins, changes in protein tyr phosphorylation, etc.) In other tissues, VLK participates in hedgehog signaling - is that happening here?

Additionally, the temporal and spatial expression patterns of VLK are also quite complex such that it is difficult to tease out what the authors are really asking/investigating. VLK is highly regulated during development being expressed in high levels in embryonic hepatocytes then shifting to a bile duct distribution in adults. Yet they chose a hepatocyte-specific KO approach and are studying

© 2022. Published by The Company of Biologists under the terms of the Creative Commons Attribution License (https://creativecommons.org/licenses/by/4.0/).

changes in perivascular fibrosis and inflammation in the adult where the bile duct localization and expression of VLK are not changed. How do the phenotypes relate to a hepatocyte specific KO? Are they studying liver progenitor cells that now lack VLK and how that affects tissue repair? Why would that affect collagen deposition when stellate cells presumably still express VLK? Is it really a paracrine mechanism as hypothesized? Does the conditioned medium from the KO de-differentiated hepatocytes alter liver non-parenchymal cell phenotypes? Via what mechanism?

Clearly, this is more than can be addressed in a single manuscript, but the authors are not yet to the mechanistic studies. The studies as presented now may be better suited for a journal more specifically targeting translational/clinical liver scientists or other GI researchers.

<u>Reviewer 2</u>

Advance summary and potential significance to field

The work by Pantasis et al. addresses the role of VLK (Vertebrate Lonesome Kinase) in the liver by specific knock-out in hepatocytes. VLK is a unique, but poorly understood, secreted tyrosine kinase with considerable potential for influencing the cellular microenvironment, tissue homeostasis and tissue remodelling. The study describes the expression of Vlk in the liver during development – predominantly in ductular, but also in immature hepatocytic epithelia. A series of interesting and novel in vitro experiments are performed, using organoids, to demonstrate a role for Vlk in hepatocytes that are expanded as ductular progenitor cells. In this dedifferentiated state, Vlk appears to limit epithelial expansion by altering/conditioning the secretome, and by influencing the behaviour of fibroblasts, which are important regulators of liver regeneration and repair. In complementary in vivo studies, the authors also show that aged KO mice, display aberrant ductular expansion, marked steatosis, inflammation, mild cholestasis and pericentral fibrosis, highlighting a role for hepatocyte derived Vlk in maintaining liver tissue homeostasis.
These findings represent a real and significant contribution to the field. They demonstrate potential for Vlk to protect the liver from disease-relevant processes such as inflammation, fibrosis and steatosis, whilst also shedding light on basic aspects of liver progenitor cell expansion during injury. The manuscript is very well written and concise, the figures are well organized and clearly presented. The work combines an array of technically cutting edge approaches (organoid culture and RNAscope) that provide a detailed, if not exhaustive, first description of the role Vlk in liver.

Comments for the author

- Additional minor comments:
- In the results section, the authors identify VLK expression in what they refer to as “clustered cells” (p6 and Figure 1b) – these should be indicated by arrows in figure 1.
- In continuation from the above comment, the embryonic liver typically contains clusters of haemopoietic cells and clustered structures in portal tracts that go on to form ducts, but hepatocytes do not usually form discrete clusters. In the text (p6) the authors suggest, “based on distribution and [presumably nuclear] morphology”, that “most of the VLK positive cells are hepatocytes”. This could have been easily tested/corroborated by co-staining for hepatocyte markers (such as Hnf4a). Did the authors try such an approach? It would be useful to know the criteria used to back up their hypothesis.
- The portal/central vein should be clearly annotated in all of the figures to help orient the reader. For example, it is unclear in figures 4 A-C, G and Figure 5B which area of the liver the images are centred upon.
- Typographic error Page 3 – Line 3, “resident” – should be “residents”, ?
- Error detected – Page 13 line 18 “with CCl_4 or oil (vehicle) (Fig. 6D)" – should be "(Fig. S6D)"?
- Figure 5B, D – It is difficult for the reader to understand exactly what is being quantified in this figure. Can an image of the bridge phenotype be included to help, or can it be referred to or highlighted in figure 5B?
Bridging fibrosis between portal tracts is normally a measure of the severity of fibrosis. Are the authors saying that the bridges are fewer, so therefore there is less fibrosis, or that the bridges are larger/more connected so there is more fibrosis? It is important to clarify this point given that these are the key data in this figure describing the subtle difference in the injury response between Ctrl and KO mice.

© 2022. Published by The Company of Biologists under the terms of the Creative Commons Attribution License (https://creativecommons.org/licenses/by/4.0/).

Reviewer 3

Advance summary and potential significance to field

The manuscript reports that vertebrate lonesome kinase (VLK) is highly expressed in neonatal but not adult mouse hepatocytes. The mouse with hepatocyte specific ablation of VLK appears normal. However, it gradually develops liver steatosis and have expansion of liver progenitor cells. In addition, charllenging the VLK null mice with CCl4 induces stronger damages than in control mice. the results suggest a protective function of hepatocyte-derived VLK during homeostasis, aging, and liver injury.
Overall, the data are solid and support the conclusion, although lack in-depth mechanistic studies.

Comments for the author

Major points: Fig. 2g needs to have adding VLK protein in the ko cell conditioned medium to proof that the activity is VLK dependent.
Fig. 2F need to include a control showing whether addition of VLK compromises the proliferation stimulated by the conditioned medium.

First revision

Author response to reviewers' comments

Response to Reviewers

Reviewer # 1 (Remarks to the Author):

This manuscript provides extensive phenotypic characterization of mice with a liver-specific knockout of vertebrate lonesome kinase (VLK)- the only know secreted tyr kinase. The phenotypes (albeit complex) would likely be interesting to those clinical/translational researchers actively studying liver disease (especially fibrosis) or liver regeneration.
This manuscript examines the mouse phenotypes of hepatocyte-specific knock out of Vertebrate lonesome Kinase (VLK). VLK is the only known secreted tyrosine kinase - an inherently interesting property – and from studies in other tissues, VLK has been shown to phosphorylate a host of proteins both in the secretory pathway and in the extracellular milieu. It has not been studied in liver – so the premise for these studies is interesting. The manuscript represents an enormous body of work both in the 5 figures within the manuscript itself and the 6 supplementary figures. The experiments were performed with rigor and the appropriate controls, statistical analysis and are informative. The results are likely quite interesting to those actively studying liver disease and regeneration and to physician scientists.

Our reply:
We thank the Reviewer for her/his positive comments. We agree that the study will be of interest to researchers working on liver disease. However, it is not a clinical study, but a functional analysis of VLK in liver homeostasis and regeneration. The liver was used as a model system to study a poorly characterized kinase, and our results provide the first information on the function of VLK in this organ. Therefore, we believe that it is also of high interest to cell biologists that study mechanistic questions.

The concern is that the studies may not appropriate for the JCS readership where mechanistic, cell biological studies are highlighted. The studies described in this manuscript are largely descriptive in nature and reveal phenotypes (many of which are interesting, but very complex) without any mechanistic details of VLK function. For example, the title of the paper states VLK modulates the hepatocyte secretome. However, the experiments shown in Fig. 2 to support that statement are indirect - no alterations in the secretome were specifically examined (e.g., the profile of secreted proteins, changes in protein tyr phosphorylation, etc.).

© 2022. Published by The Company of Biologists under the terms of the Creative Commons Attribution License (https://creativecommons.org/licenses/by/4.0/).

Our reply:
We agree with the reviewer that the observed phenotypes are complex, and therefore, the mechanistic interpretation of the data is not trivial. However, we believe that it does contain important mechanistic *in vivo* and *in vitro* data. First, the knockout was specifically induced in hepatocytes, which allowed us to determine the contribution of this specific cell type to the phenotype. Second, the *in vivo* data are complemented by functional *in vitro* studies supporting a role for secreted VLK in modulating cell proliferation through a paracrine mechanism. Additional mechanistic studies have been included into the revised manuscript (see below).

A full proteomics analysis of the secretome complemented by a phospho-proteomics screen to identify direct VLK substrates is a very appealing avenue for follow-up studies. However, we believe that this is beyond the scope of a first manuscript on VLK function in the liver. Moreover, the technical challenges related to obtaining enough starting material from reprogrammed hepatocytes for a meaningful analysis were not compatible with the limited time of a revision.

Nevertheless, we attempted to address the important reviewer's comment during the limited time frame available for the revision by performing a proteomics analysis of whole cell lysates (WCL) of cultured reprogrammed Ctrl and VLK KO hepatocytes. Among the differentially abundant proteins we focused on those with a reported signal sequence. This approach led to the identification of alpha-fetoprotein (AFP), a known marker for liver injury and hepatocellular carcinoma, which is secreted by hepatic progenitor cells , (Kuhlmann and Peschke, 2006, Alison et al., 2009). Increased AFP abundance was confirmed in the secretome of reprogrammed VLK KO cells and in liver tissue of aged and CCl_4-treated VLK KO mice. Importantly, AFP has been shown to increase proliferation of fibroblasts (Li et al., 2002). While it is unlikely that one single secreted factor mediates all the phenotypic abnormalities that we identified in the VLK-deficient mice, the upregulation of AFP could at least in part explain our findings. The data also demonstrate that the secretome of VLK-deficient, reprogrammed hepatocytes indeed differs in its composition from the secretome of reprogrammed control cells. These new data are now shown in Fig. 6 and Fig. S6.

In other tissues, VLK participates in hedgehog signaling – is that happening here? Additionally, the temporal and spatial expression patterns of VLK are also quite complex such that it is difficult to tease out what the authors are really asking/investigating. VLK is highly regulated during development being expressed in high levels in embryonic hepatocytes then shifting to a bile duct distribution in adults. Yet they chose a hepatocyte-specific KO approach and are studying changes in perivascular fibrosis and inflammation in the adult where the bile duct localization and expression of VLK are not changed. How do the phenotypes relate to a hepatocyte specific KO? Are they studying liver progenitor cells that now lack VLK and how that affects tissue repair?

Our reply:
To address the first question of the reviewer, we analyzed the expression of the classical hedgehog target genes *Gli1* and/or *Ccnd1* in Ctrl and VLK KO primary hepatocytes, reprogrammed cells and mouse tissue. We did not detect a significant difference between genotypes in any of the conditions. These data strongly suggest that in our liver model, loss of VLK does not impact hedgehog signaling. These new data have been included in Fig. S1J and Fig. S4G and H.

The described temporal and spatial expression pattern of VLK is indeed very complex. Our initial data showed VLK expression in hepatocytes and in bile duct cells at different developmental stages. Since the Albumin-Cre mice allow hepatocyte-specific deletion of the gene of interest shortly before birth, we used this approach to determine whether the early loss of VLK in hepatocytes (when VLK expression is high in this cell-type) affects the postnatal development of the liver. The phenotypic abnormalities that we already observed in young hepatocyte-specific *Pkdcc* knockout mice indeed point into this direction. In addition, *Pkdcc* expression is still detectable at the RNA level in hepatocytes of adult KO mice, which is likely to lead to low levels of VLK protein, which may be below detection level in immunofluorescence studies. Nevertheless, low levels of an active enzyme can still be biologically important. While our results do not allow to distinguish between roles of VLK during late liver development *vs.* homeostasis in adult mice, we can conclude that VLK in hepatocytes is functionally relevant.

© 2022. Published by The Company of Biologists under the terms of the Creative Commons Attribution License (https://creativecommons.org/licenses/by/4.0/).

To further address the comments of the reviewer, we also included new data from mouse model with an inducible CK19-Cre$^{(ERT)}$ driven deletion of *Pkdcc* in biliary epithelial cells. Surprisingly, we did not find obvious histological abnormalities in these mice - neither in unchallenged young or aged mice nor upon chronic CCl4 injury. Although dietary challenges, such as DDC or CDE diets (Akhurst et al., 2001, Michalopoulos, 2011, Pose et al., 2019), may reveal a role for VLK in cholangiocytes in the future, our results demonstrate that despite its strong expression, an acute loss of *Pkdcc* in cholangiocytes does not affect liver homeostasis. These new data are now reported in Fig. S3I-M, Fig. S4I and Fig. S5H.

Why would that affect collagen deposition when stellate cells presumably still express VLK? Is it really a paracrine mechanism as hypothesized? Does the conditioned medium from the KO de-differentiated hepatocytes alter liver non-parenchymal cell phenotypes? Via what mechanism? Clearly, this is more than can be addressed in a single manuscript, but the authors are not yet to the mechanistic studies. The studies as presented now may be better suited for a journal more specifically targeting translational/clinical liver scientists or other GI researchers.

Our reply:
As noted by the reviewer, available single cell transcriptomics data indeed suggest that stellate cells express *Pkdcc* and we confirmed the expression of this gene in non-parenchymal cells of the liver (Fig. 3C). It is unclear, however, whether stellate cell-derived VLK can compensate for the loss of hepatocyte-specific VLK, and our data argue against this possibility. Several examples in the literature report severe phenotypes in mice lacking a secreted protein in a specific cell type, although neighboring cells in the tissue still express it. An example are the different skin/wound healing phenotypes observed in mice lacking the growth factor VEGF in keratinocytes vs. myeloid cells (Elias et al., 2008, Willenborg et al., 2012). Determination of cell type-specific functions of VLK in the liver is an interesting avenue for future studies, and we now mention this in the Discussion (page 19).

We do believe that our extensive *in vitro* co-culture studies support a paracrine effect of the secretome, and we propose AFP (Fig. 6 and Fig. S6) as one of the potential mediators. Our data indeed demonstrate that the secretome of reprogrammed VLK KO cells positively impacts the proliferation of fibroblasts, and literature suggests that AFP may be involved in this regulation (Li et al., 2002). Additional studies report that secreted AFP can regulate cell growth (Li et al., 2002, Zhang et al., 2012) and inhibit differentiation of dendritic cells (Wang and Wang, 2018).

Taken together, we believe that the new data have strengthened the functional aspects of the manuscript.

Reviewer 2

The work by Pantasis et al. addresses the role of VLK (Vertebrate Lonesome Kinase) in the liver by specific knock-out in hepatocytes. VLK is a unique, but poorly understood, secreted tyrosine kinase with considerable potential for influencing the cellular microenvironment, tissue homeostasis and tissue remodelling. The study describes the expression of Vlk in the liver during development – predominantly in ductular, but also in immature hepatocytic epithelia. A series of interesting and novel in vitro experiments are performed, using organoids, to demonstrate a role for Vlk in hepatocytes that are expanded as ductular progenitor cells. In this dedifferentiated state, Vlk appears to limit epithelial expansion by altering/conditioning the secretome, and by influencing the behaviour of fibroblasts, which are important regulators of liver regeneration and repair. In complementary in vivo studies, the authors also show that aged KO mice, display aberrant ductular expansion, marked steatosis, inflammation, mild cholestasis and pericentral fibrosis, highlighting a role for hepatocyte derived Vlk in maintaining liver tissue homeostasis. These findings represent a real and significant contribution to the field. They demonstrate potential for Vlk to protect the liver from disease-relevant processes such as inflammation, fibrosis and steatosis, whilstalso shedding light on basic aspects of liver progenitor cell expansion during injury. The manuscript is very well written and concise, the figures are well organized and clearly presented. The work combines an array of technically cutting edge approaches (organoid culture and RNAscope) that provide a detailed, if not exhaustive, first description of the role Vlk in liver.

© 2022. Published by The Company of Biologists under the terms of the Creative Commons Attribution License (https://creativecommons.org/licenses/by/4.0/).

Our reply:

We thank the reviewer for the positive comments and the important suggestions.

- *In the results section, the authors identify VLK expression in what they refer to as "clustered cells" (p6 and Figure 1b) – these should be indicated by arrows in figure 1.*

Our reply: Arrows were included in Figure 1B as suggested.

- *In continuation from the above comment, the embryonic liver typically contains clusters of haemopoietic cells and clustered structures in portal tracts that go on to form ducts, but hepatocytes do not usually form discrete clusters. In the text (p6) the authors suggest, "based on distribution and [presumably nuclear] morphology", that "most of the VLK positive cells are hepatocytes". This could have been easily tested/corroborated by co-staining for hepatocyte markers (such as Hnf4a). Did the authors try such an approach? It would be useful to know the criteria used to back up their hypothesis.*

Our reply: We addressed this concern by staining serial liver sections with antibodies against VLK and the hepatocyte marker albumin. A co-staining approach did not work due to antibody incompatibility. The new data included in Fig. S1A show that most of the VLK-positive cell clusters also express albumin, strengthening our initial interpretation.

- *The portal/central vein should be clearly annotated in all of the figures to help orient the reader. For example, it is unclear in figures 4 A-C, G and Figure 5B which area of the liver the images are centred upon.*
- *Typographic error Page 3 – Line 3, "resident" – should be "residents", ?*
- *Error detected – Page 13 line 18 "with CCl4 or oil (vehicle) (Fig. 6D)" – should be "(Fig. S6D)"?*

Our reply: The figures have been modified according to the reviewer`s suggestions. Typos have been corrected.

- *Figure 5B, D – It is difficult for the reader to understand exactly what is being quantified in this figure. Can an image of the bridge phenotype be included to help, or can it be referred to or highlighted in figure 5B? Bridging fibrosis between portal tracts is normally a measure of the severity of fibrosis. Are the authors saying that the bridges are fewer, so therefore there is less fibrosis, or that the bridges are larger/more connected so there is more fibrosis? It is important to clarify this point given that these are the key data in this figure describing the subtle difference in the injury response between Ctrl and KO mice.*

Our reply: We thank the reviewer for noticing this ambiguity. The initially reported analysis focused on the description of the observed phenotype. Since, however, fibrosis staging and quantification is of major importance in human liver disease, we now include a scoring of fibrosis severity in Fig. 5E that was performed by an experienced liver pathologist. According to *Bedossa P, Poynard T (Bedossa and Poynard, 1996)- An algorithm for the grading of activity in chronic hepatitis C. The METAVIR Cooperative Study,* the well-established METAVIR score discriminates between bridging fibrosis with few septae versus more severe fibrosis with many septae. To increase data quality, we have repeated all Sirius red stainings. The new analysis reveals a clear trend towards more and broader septae, and therefore more severe fibrosis, in VLK KO animals.

Reviewer 3

The manuscript reports that vertebrate lonesome kinase (VLK) is highly expressed in neonatal but not adult mouse hepatocytes. The mouse with hepatocyte specific ablation of VLK appears normal. However, it gradually develops liver steatosis and have expansion of liver progenitor cells. In addition, challenging the VLK null mice with CCl4 induces stronger damages than in control mice. The results suggest a protective function of hepatocyte-derived VLK during homeostasis, aging, and liver injury. Overall, the data are solid and support the conclusion, although lack in-depth mechanistic studies.

Our reply: We thank reviewer for the positive comments and important suggestions. Regarding the mechanistic studies, we kindly refer to our reply to reviewer 1 and to the new data that we

© 2022. Published by The Company of Biologists under the terms of the Creative Commons Attribution License (https://creativecommons.org/licenses/by/4.0/).

have included into the manuscript in response to these criticisms.

Major points: Fig. 2g needs to have adding VLK protein in the ko cell conditioned medium to proof that the activity is VLK dependent. Fig. 2F need to include a control showing whether addition of VLK compromises the proliferation stimulated by the conditioned medium.

Our reply: We aimed to address this point by purchasing commercially available recombinant VLK protein from Abcam (ab131683). Unfortunately, this specific product is not biologically active as confirmed by the vendor. The time available for the revisions and the significant number of other experiments performed did not allow us to express and purify biologically active VLK on our own. We would also like to mention that addition of recombinant VLK may not be conclusive, since VLK plays a role in the secretory pathway ((Bordoli et al., 2014)). These intracellular effects would not be affected by addition of recombinant protein to the cell supernatant. Nevertheless, we agree with the Reviewer that biologically active VLK is definitely a helpful tool for future *in vitro* applications.

Most importantly, we have addressed the concern of the reviewer regarding the lack of in-depth mechanistic studies by performing a quantitative proteomics experiments and follow-up studies (Fig. 6, Fig. S6) and by generation and characterization of a mouse model that lacks VLK in cholangiocytes (Fig. S3I-M, Fig. S4I and Fig. S5H)

Reference List:

AKHURST, B., CROAGER, E. J., FARLEY-ROCHE, C. A., ONG, J. K., DUMBLE, M. L., KNIGHT, B. & YEOH, G. C. 2001. A modified choline-deficient, ethionine-supplemented diet protocol effectively induces oval cells in mouse liver. *Hepatology,* 34, 519-22.

ALISON, M. R., ISLAM, S. & LIM, S. M. 2009. Cell therapy for liver disease. *Curr Opin Mol Ther,* 11, 364- 74.

BEDOSSA, P. & POYNARD, T. 1996. An algorithm for the grading of activity in chronic hepatitis C. The METAVIR Cooperative Study Group. *Hepatology,* 24, 289-93.

BORDOLI, M. R., YUM, J., BREITKOPF, S. B., THON, J. N., ITALIANO, J. E., JR., XIAO, J., WORBY, C., WONG, S. K., LIN, G., EDENIUS, M., KELLER, T. L., ASARA, J. M., DIXON, J. E., YEO, C. Y. & WHITMAN, M. 2014. A secreted tyrosine kinase acts in the extracellular environment. *Cell,* 158, 1033-1044.

ELIAS, P. M., ARBISER, J., BROWN, B. E., ROSSITER, H., MAN, M. Q., CERIMELE, F., CRUMRINE, D., GUNATHILAKE, R., CHOI, E. H., UCHIDA, Y., TSCHACHLER, E. & FEINGOLD, K. R. 2008. Epidermal vascular endothelial growth factor production is required for permeability barrier homeostasis, dermal angiogenesis, and the development of epidermal hyperplasia: implications for the pathogenesis of psoriasis. *Am J Pathol,* 173, 689-99.

KUHLMANN, W. D. & PESCHKE, P. 2006. Hepatic progenitor cells, stem cells, and AFP expression in models of liver injury. *Int J Exp Pathol,* 87, 343-59.

LI, M. S., LI, P. F., YANG, F. Y., HE, S. P., DU, G. G. & LI, G. 2002. The intracellular mechanism of alpha-fetoprotein promoting the proliferation of NIH 3T3 cells. *Cell Res,* 12, 151-6.

MICHALOPOULOS, G. K. 2011. Liver regeneration: alternative epithelial pathways. *Int J Biochem Cell Biol,* 43, 173-9.

POSE, E., SANCHO-BRU, P. & COLL, M. 2019. 3,5-Diethoxycarbonyl-1,4-Dihydrocollidine Diet: A Rodent Model in Cholestasis Research. *Methods Mol Biol,* 1981, 249-257.

WANG, X. & WANG, Q. 2018. Alpha-Fetoprotein and Hepatocellular Carcinoma Immunity. *Can J Gastroenterol Hepatol,* 2018, 9049252.

WILLENBORG, S., LUCAS, T., VAN LOO, G., KNIPPER, J. A., KRIEG, T., HAASE, I., BRACHVOGEL, B., HAMMERSCHMIDT, M., NAGY, A., FERRARA, N., PASPARAKIS, M. & EMING, S. A. 2012. CCR2 recruits an inflammatory macrophage subpopulation critical for angiogenesis in tissue repair. *Blood,* 120, 613-25.

ZHANG, W., LI, W., LIU, B., WANG, P., LI, W. & ZHANG, H. 2012. Efficient generation of functional hepatocyte-like cells from human fetal hepatic progenitor cells in vitro. *J Cell Physiol,* 227, 2051- 8.

© 2022. Published by The Company of Biologists under the terms of the Creative Commons Attribution License (https://creativecommons.org/licenses/by/4.0/).

Second decision letter

MS ID#: JOCES/2021/259243

MS TITLE: Vertebrate Lonesome Kinase modulates the hepatocyte secretome to prevent perivascular liver fibrosis and inflammation

AUTHORS: Sophia Pantasis, Juliane Friemel, Salome Bruetsch, Zehan Hu, Sabrina Krautbauer, Gerhard Liebisch, Joern Dengjel, Achim Weber, Sabine Werner, and Mattia Bordoli
ARTICLE TYPE: Research Article

I am happy to tell you that your manuscript has been accepted for publication in Journal of Cell Science, pending standard ethics checks.

Reviewer 1

Advance summary and potential significance to field

This manuscript examines the mouse phenotypes of hepatocyte-specific knock out of Vertebrate Lonesome Kinase (VLK). VLK is the only known secreted tyrosine kinase
- an inherently interesting property – and from studies in other tissues, VLK has been shown to phosphorylate proteins both in the secretory pathway and in the extracellular milieu. It has not been studied in liver – so the premise for these studies is interesting. In the end, the authors provide ample evidence that secreted VLK is hepatoprotective against perivascular fibrosis and inflammation by altering the hepatic secretome.

Comments for the author

The authors have been very responsive to the concerns written in my manuscript original review. They have performed multiple additional experiments, many of which were not trivial, to bolster their original results and support their conclusions. This manuscript represents an enormous body of work and adds to our understanding of the understudied VLK.

Reviewer 2

Advance summary and potential significance to field

./.

Comments for the author

I am satisfied that the authors have addressed all of my comments.

Reviewer 3

Advance summary and potential significance to field

/

Comments for the author

I have no additional comments.

© 2022. Published by The Company of Biologists under the terms of the Creative Commons Attribution License (https://creativecommons.org/licenses/by/4.0/).
